# Role of Anionic Backbone in NHC‐Stabilized Coinage Metal Complexes: New Precursors for Atomic Layer Deposition**

**DOI:** 10.1002/chem.202103798

**Published:** 2022-02-15

**Authors:** Nils Boysen, Anish Philip, Detlef Rogalla, Maarit Karppinen, Anjana Devi

**Affiliations:** ^1^ Inorganic Materials Chemistry (IMC) Ruhr University Bochum 44801 Bochum Germany; ^2^ Department of Chemistry and Materials Science Aalto University P.O. Box 16100 00076 Espoo Finland; ^3^ RUBION Ruhr University Bochum 44801 Bochum Germany

**Keywords:** atomic layer deposition, carbene ligands, coinage metals, precursors, X-ray diffraction

## Abstract

Cu and Ag precursors that are volatile, reactive, and thermally stable are currently of high interest for their application in atomic‐layer deposition (ALD) of thin metal films. In pursuit of new precursors for coinage metals, namely Cu and Ag, a series of new N‐heterocyclic carbene (NHC)‐based Cu^I^ and Ag^I^ complexes were synthesized. Modifications in the substitution pattern of diketonate‐based anionic backbones led to five monomeric Cu complexes and four closely related Ag complexes with the general formula [M(^*t*Bu^NHC)(R)] (M=Cu, Ag; ^*t*Bu^NHC=1,3‐di‐*tert*‐butyl‐imidazolin‐2‐ylidene; R=diketonate). Thermal analysis indicated that most of the Cu complexes are thermally stable and volatile compared to the more fragile Ag analogs. One of the promising Cu precursors was evaluated for the ALD of nanoparticulate Cu metal deposits by using hydroquinone as the reducing agent at appreciably low deposition temperatures (145–160 °C). This study highlights the considerable impact of the employed ligand sphere on the structural and thermal properties of metal complexes that are relevant for vapor‐phase processing of thin films.

## Graphical abstract

**In pursuit** of new precursors for the deposition of coinage metals by atomic layer deposition (ALD), a series of new N‐heterocyclic carbene (NHC)‐based Cu^I^ and Ag^I^ complexes has been synthesized, analyzed, and evaluated for their potential use in ALD. One precursor was found to be particularly useful for the deposition of metallic copper when using hydroquinone as the reducing agent in a thermally driven ALD process.

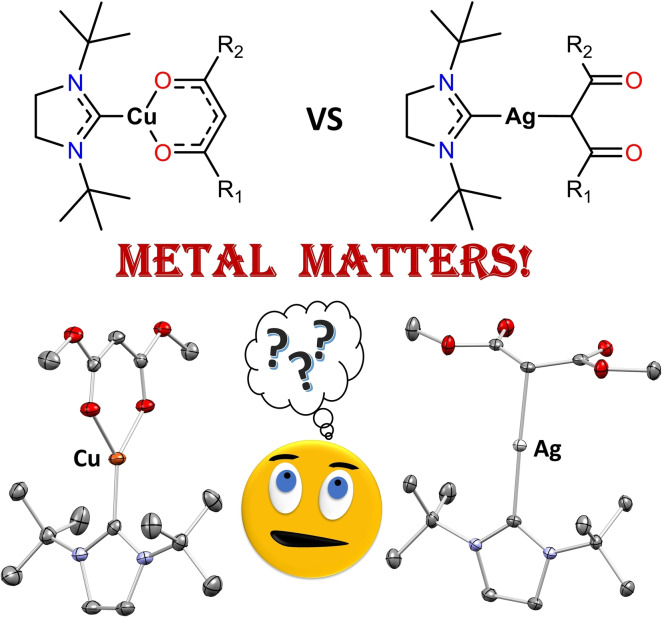

## Introduction

Metalorganic Cu^I^ and Ag^I^ complexes have remarkable similarities in their bonding behavior, structures and physico‐chemical properties.^[1]^ Their rich chemistry has been exploited in numerous applications in the past, which range from applications in homogeneous catalysis,^[2]^ potential anticancer agents to precursors for vapor phase deposition processes delivering Cu‐ and Ag‐containing thin films.[^3^, ^4^] In particular metallic Cu and Ag thin films are highly interesting for (opto)electronic applications owing to the appreciably low electrical bulk resistivities of Cu (0.168 μΩ cm) and Ag (0.159 μΩ cm), the latter being the lowest achievable value among all metals.^[5]^ The low resistivity is also important in applications such as Cu interconnects in integrated circuits (ICs) or ultra‐thin Ag layers as transparent electrodes for solar cells and light‐emitting devices.[^6^, ^7^] In these applications, a thin layer of Cu or Ag is needed in order to ensure a well performing device, which can be deposited by vapor‐phase deposition techniques such as physical vapor deposition (PVD), chemical vapor deposition (CVD) or atomic layer deposition (ALD).[^8^, ^9^] The ALD technique benefits from a high thin film homogeneity on large area surfaces, exceptional film conformality and film thickness control on the atomic level, as the growth of the film proceeds layer‐by‐layer. These characteristics can only evolve if the precursor molecules, typically a metalorganic or organometallic compound, chemisorbs to the functional surface groups of the substrate in a self‐saturated manner.^[10]^ To achieve self‐saturation, the precursor not only has to be highly reactive towards the functional groups on the surface of the substrate, but must also feature a high stability within the employed process temperature regime.^[11]^ Additionally, a high volatility of the precursor at low temperatures is beneficial to enhance the temperature regime in which the self‐limiting growth of thin films can be achieved.

For the formation of Cu and Ag containing thin films by ALD, different classes of precursors have been employed in the past. The Cu precursor classes can be divided in two groups based on the oxidation state, namely Cu^I^ and Cu^II^ precursors, whereas for Ag only Ag^I^ precursors are known, due to the instability of divalent Ag. For the sake of comparability, only selected Cu^I^ and Ag^I^ precursors are discussed here; a more comprehensive overview of the Cu^I^ and Cu^II^ precursors for ALD and CVD is found in the recent review article by Hagen et al.^[12]^

All‐nitrogen‐coordinated precursors in the form of amidinates with different substitution patterns have been proposed by Lim et al.^[13]^ and Li et al.^[14]^ which include the dinuclear complexes [Cu(^*i*^PrAMD)]_2_ and [Cu(^*s*^BuAMD)]_2_. Both precursors are reactive, volatile and thermally stable and thus successfully used in combination with H_2_ gas or H_2_ plasma for the ALD growth of Cu metal films with deposition temperatures as low as 50 °C.^[15]^ A similar structure is also known for Ag^I^, although a mixture of dimers and trimers [Ag(^*i*^PrAMD)]_2/3_ was obtained according to Lim et al.^[13]^ This compound has not so far been challenged as an ALD precursor despite its promising thermal properties.

In order to obtain mononuclear complexes, it is beneficial to employ neutral ligands that strongly bind to the Cu or Ag metal and prevent oligomerization.^[16]^ In this way, the central metal atom is not only electronically stabilized by σ‐electron donation from the neutral ligand to the metal and π‐electron backdonation from the metal to the ligand, but might also be sterically forced into a mononuclear state.^[17]^ Additionally, the heteroleptic coordination environment can be systematically fine‐tuned by either exchanging and altering the neutral (alkenes, alkynes, phosphines, carbenes) or the anionic ligand (diketonates, amides, diketiminates) which naturally results in a higher number of different potential precursors that can be obtained. Alkenes as neutral ligands have been successfully used for Cu^I^ precursors in the case of [Cu(hfac)(vtmos)] (hfac: hexafluoroacetylacetone, vtmos: vinyltrimethoxysilane) by Moon et al.^[18]^ and [Cu(dki)(vtms)] (dki: diketiminate, vtms: vinyltrimethylsilane) by Park et al.^[19]^ who employed these precursors in combination with H_2_ plasma or SiH_2_Et_2_ for the ALD of Cu metal films. In the latter study, the substitution pattern of the anionic diketiminate was systematically fine‐tuned and precursors with considerably enhanced volatility compared to the dinuclear complex [Cu(^*i*^PrAMD)]_2_ were obtained while at the same time sacrificing on the thermal stability of the complexes. The high volatility and reactivity of complexes towards reducing agents enabled the growth of Cu films at the low deposition temperature of 120 °C. Enhancement of the thermal stability of these alkene coordinated complexes was investigated by Norman et al. who developed bridged anionic‐neutral ligands, which resulted in the volatile precursor class [Cu(kim‐vtms)] (kim: ketoiminate) and further enabled the growth of Cu films in the temperature range 130–200 °C using H_2_ gas as the reductant.^[20]^ Phosphines were used as neutral ligands for Cu^I^ and especially for Ag^I^ precursors using different anionic backbones such as diketonates or pivaloyl derivatives. [Cu(acac)(P^n^Bu_3_)] (acac: acetylacetonate) was employed by Waechtler et al. for the ALD of Cu oxide layers which resulted in Cu seed layers after thermal reduction of the oxide.[^21^, ^22^] Very similar Ag precursors have extensively been used for the growth of Ag metal films by ALD. [Ag(fod)(PEt_3_)] (fod: 1,1,1,2,2,3,3‐heptafluoro‐7,7‐dimethyloctane‐4,6‐dione) was introduced as precursor by Kariniemi et al.^[23]^ for the deposition of Ag films at low temperatures of 120 to 150 °C (H_2_ plasma) and has been extensively used in numerous ALD processes due to its superior thermal properties compared to [Ag(piv)(PEt_3_)],^[24]^ or [Ag(hfac)(cod)] (cod: cyclooctadiene).[^25^, ^26^, ^27^, ^28^, ^29^] The deposition of metal films was achieved using H_2_ plasma and organic reducing agents such as hydrazines, propanol and boranes. A rather new development in this field is the employment of N‐heterocyclic carbenes (NHCs) as highly stable neutral ligands for Cu^I^ and Ag^I^ precursors. Initial studies by Coyle et al. revealed the promising characteristics of NHCs combined with trimethylsilylamides as ligand partners in Cu^I^ complexes delivering highly reactive and volatile precursors, for example, [Cu(^*i*Pr^NHC)(hmds)] (hmds: hexamethyldisilazide, ^*i*Pr^NHC=1,3‐di‐iso‐propyl‐imidazolin‐2‐ylidene) for Cu metal ALD at deposition temperatures of 225 °C.^[30]^ The same concept was later adopted in our recent studies for the spatial ALD of Cu and Ag metal films using [Cu(^*t*Bu^NHC)(hmds)] and [Ag(^*t*Bu^NHC)(hmds)] and H_2_ plasma at low deposition temperatures of 100 and 120 °C, respectively, while also comparing the similarities and differences between the nature, thermal stability and reactivity of the Cu and Ag complexes.[^31^, ^32^] While Coyle et al. investigated cyclic and acyclic carbenes with different substitution patterns for Cu complexes to assess their influence on volatility and thermal stability while keeping the anionic backbone as a constant in the respective complexes,^[33]^ we envisioned to systematically vary the anionic backbone within NHC stabilized complexes of Cu and Ag. This should not only increase the understanding of the influence of the anionic backbone variation on parameters like thermal stability, volatility and reactivity but also possibly eliminate unwanted elements in the ligand sphere that are prone to incorporate into the thin films such as fluorine, phosphorous or silicon which is one of the drawbacks of the most established Cu^I^ and Ag^I^ precursors. It should be further highlighted that there is still a considerable lack of suitable Ag precursors for ALD that combine a high volatility with a high thermal stability and reactivity, which underlines the importance of rational ligand design and choice to directly influence the physico‐chemical properties as desired for the applicability of these Ag precursors in ALD. In this context, we synthesized new Cu^I^ and Ag^I^ complexes based on NHCs as the neutral ligand and different diketonates as the anionic ligand with the general formula [M(^*t*Bu^NHC)(R)] (R=diketonates), while keeping the NHC ligand as a constant to enable systematic analysis and evaluation of the complexes and test their suitability for ALD processes. Finally, one representative Cu precursor from the series developed was tested in actual proof‐of‐principle vapor‐phase deposition experiments for the formation of nanoparticulate metallic Cu deposits using hydroquinone as the reducing agent. The parent study sets a new milestone in the understanding of Cu and Ag precursor chemistry in a bid to find superior potential precursors for the ALD of Cu and Ag metal thin films.

## Results and Discussion

### Precursor synthesis

**Scheme 1:**
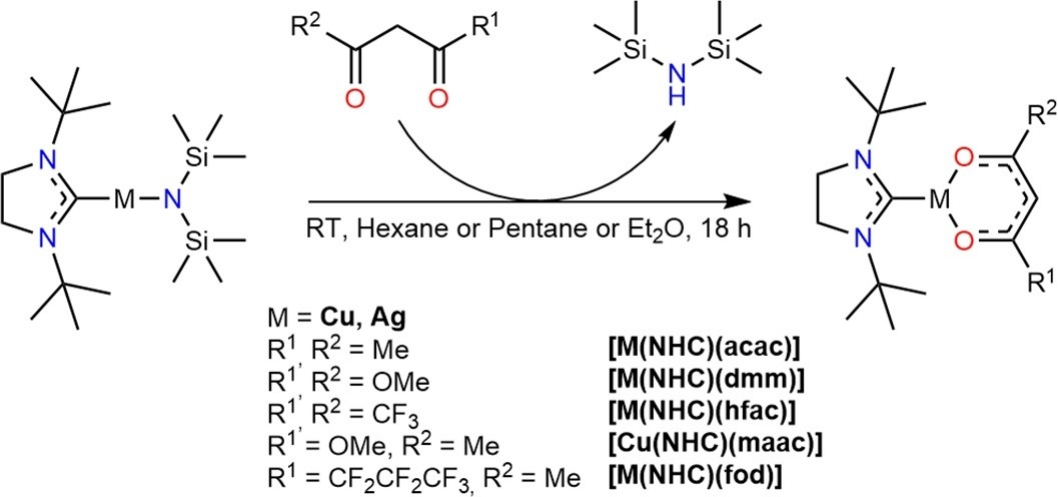
Schematic illustration of the reaction pathway for the synthesis of the desired diketonate‐based complexes.

Five different target complexes for each metal (Cu and Ag) with incremental differences in their anionic diketonate ligand systems have been chosen for synthesis. The endocyclic substituents within the anionic backbone were selected according to steric and electronic parameters: Fluorinated diketonates such as 1,1,1,2,2,3,3‐heptafluoro‐7,7‐dimethyloctane‐4,6‐dione (fod) and hexafluoroacetylacetone resemble electron‐withdrawing and sterically demanding ligands, while acetylacetone (acac), methylacetoacetate (maac), and dimethylmalonate (dmm) introduce ligands with a higher electron‐density and rather small endocyclic bulk that can interact with the central metal ion. The synthesis of the target complexes proceeded in one step with ligand‐exchange from the starting material [M(^*t*Bu^NHC)(hmds)] (M=Cu, Ag, ^*t*Bu^NHC=1,3‐di‐*tert*‐butyl‐imidazolin‐2‐ylidene) and the corresponding diketonate through an in‐situ deprotonation (Scheme 1). Due to the higher basicity of the M−N bond within the [M(^*t*Bu^NHC)(hmds)] complex in comparison to the M−O bond in the [M(^*t*Bu^NHC)(diketonate)], an in‐situ deprotonation of the diketonate is the driving force of the reaction and stabilizes the product formation. This is highlighted by the high yields (70–90 %) obtained for most of the Cu complexes, for example, [Cu(^*t*Bu^NHC)(acac)], [Cu(^*t*Bu^NHC)(fod)], [Cu(^*t*Bu^NHC)(hfac)] and [Cu(^*t*Bu^NHC)(dmm)] after crystallization directly from the reaction mixture. The batch size of the synthesis was successfully upscaled to a 3 g scale for [Cu(^*t*Bu^NHC)(acac)] and [Cu(^*t*Bu^NHC)(fod)] while retaining the high yields of 89 and 91 %, respectively. Sublimation of both complexes could be achieved at 100 °C at a pressure of 0.2 mbar.

Interestingly, the yield for the corresponding Ag complexes [Ag(^*t*Bu^NHC)(fod)] and [Ag(^*t*Bu^NHC)(hfac)] is reasonably high (89 and 61 %), while for [Ag(^*t*Bu^NHC)(acac)], [Ag(^*t*Bu^NHC)(dmm)] the yields are only poor (17 and 35 %). This might be explained by the poor solubility of these complexes in nonpolar solvents like hexane, but very high solubility in slightly polar solvents such as diethyl ether which render crystallization and thus purification of the Ag complexes difficult in our experiments. The complex [Ag(^*t*Bu^NHC)(maac)] could not be isolated due to its inherently unstable nature at room‐temperature causing decomposition after isolation. Besides the complex [Ag(^*t*Bu^NHC)(maac)], all targeted complexes could be isolated by crystallization or precipitation directly from the reaction solution for further spectroscopic analysis and thermal evaluation to choose a promising precursor candidate for proof‐of‐principle ALD studies.

### Precursor analysis and evaluation

All the Cu and Ag complexes were thoroughly analyzed by spectroscopic techniques such as nuclear magnetic resonance (NMR) spectroscopy, single‐crystal X‐ray diffraction (SC‐XRD), electron‐ionization mass spectrometry (EI‐MS) and Fourier‐transformed infrared spectroscopy (FTIR) to gain information on the purity, structural integrity, and composition of the synthesized complexes. Further, to gain information on the thermal properties of the complexes, comparative thermogravimetric analysis (TGA) and vapor pressure measurements were carried out. Finally, studies were undertaken to evaluate the reactivity of the complexes towards borohydride‐based reducing agents. This should provide a complete set of data to evaluate if the complexes are promising candidates and worthwhile to be considered for the ALD of Cu and Ag metal films.

### NMR spectroscopy

**Figure 1:**
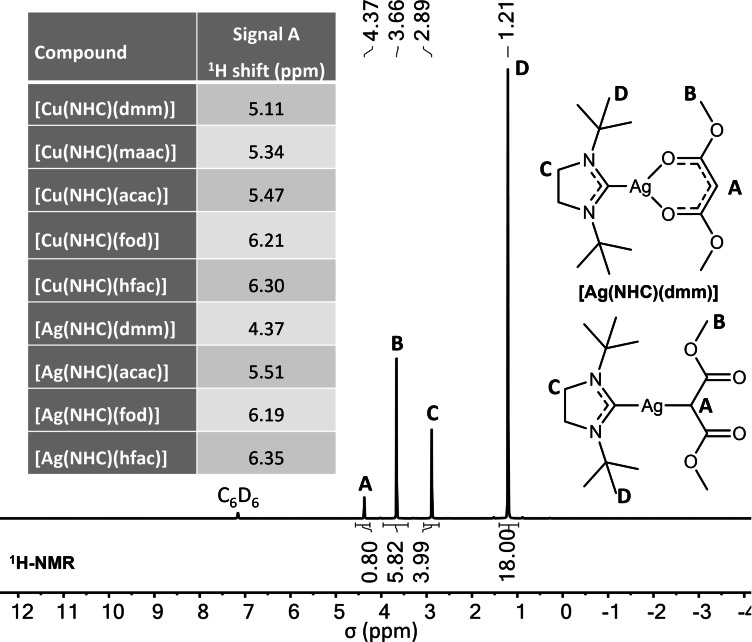
^1^H NMR spectrum of [Ag(^*t*Bu^NHC)(dmm)] with all peaks assigned to the protons within the complex. Two possible structures of [Ag(^*t*Bu^NHC)(dmm)] are drawn. A table with the chemical shifts for signal *A* seen for the different Cu and Ag complexes is illustrated as an inset.

To gain a first insight into the structure and bonding behavior of the complexes in solution, ^1^H and ^13^C NMR experiments were carried out. The proposed structure and purity of the complexes could be validated for all complexes by the proton and carbon signals in the respective NMR spectra (see the NMR section in the Supporting Information). Figure 1 shows the ^1^H NMR spectrum of [Ag(^*t*Bu^NHC)(dmm)] as an example.

All peaks seen in the ^1^H NMR spectrum of [Ag(^*t*Bu^NHC)(dmm)] can be assigned to the protons attached to the functional groups within the anionic malonate backbone and neutral NHC ligand with an integral ratio of 1 : 1 for both ligands which consequently indicates a 1 : 1 coordination of the neutral and anionic ligand. This observation can be made for all Cu and Ag complexes in this study. For a comparison of the different NMR shifts within the row of Cu and Ag complexes, signal *A*, which resembles the central proton in the anionic endocyclic diketonate ligand (O=C−C*H*−C=O), is highly interesting as it delivers a first indication of differences in the electronic nature of the different coordinating ligands. The chemical shifts for the ^1^H NMR signal *A* follow a clear trend: The ligands with higher electron density at the endocyclic backbone feature a signal shifted upfield towards lower ppm values with an overall trend dmm>maac>acac>fod>hfac, which is the same for all Cu and Ag complexes. Remarkably, the signal *A* for the complex [Ag(^*t*Bu^NHC)(dmm)] is shifted strongly to the higher field (4.37 ppm) in comparison to [Cu(^*t*Bu^NHC)(dmm)] (5.11 ppm) and thus might indicate a different bonding mode for the malonate in [Ag(^*t*Bu^NHC)(dmm)] which could be attributed to a loss of resonance stabilization combined with a change from bidentate to monodentate bonding to Ag^+^. The peak of the carbenic carbon atom in the ^13^C NMR spectrum could not be observed for all complexes due to a generally low signal intensity and strong signal splitting for Ag complexes which made a direct comparison difficult. At least for [Cu(^*t*Bu^NHC)(acac)], a signal for the carbenic carbon atom could be located in the ^13^C NMR spectrum at 203.16 ppm which is slightly shifted downfield compared to the already reported [Cu(^*t*Bu^NHC)(hmds)] complex from our earlier studies (201.6 ppm) and indicates a smaller degree of π‐back bonding from the metal to the carbene.^[32]^ Nevertheless, a more detailed insight into the bonding situation for all complexes in the solid state and a validation of the results seen in NMR studies could be gained by SC‐XRD measurements which are discussed in the next section.

### SC‐XRD

**Figure 2:**
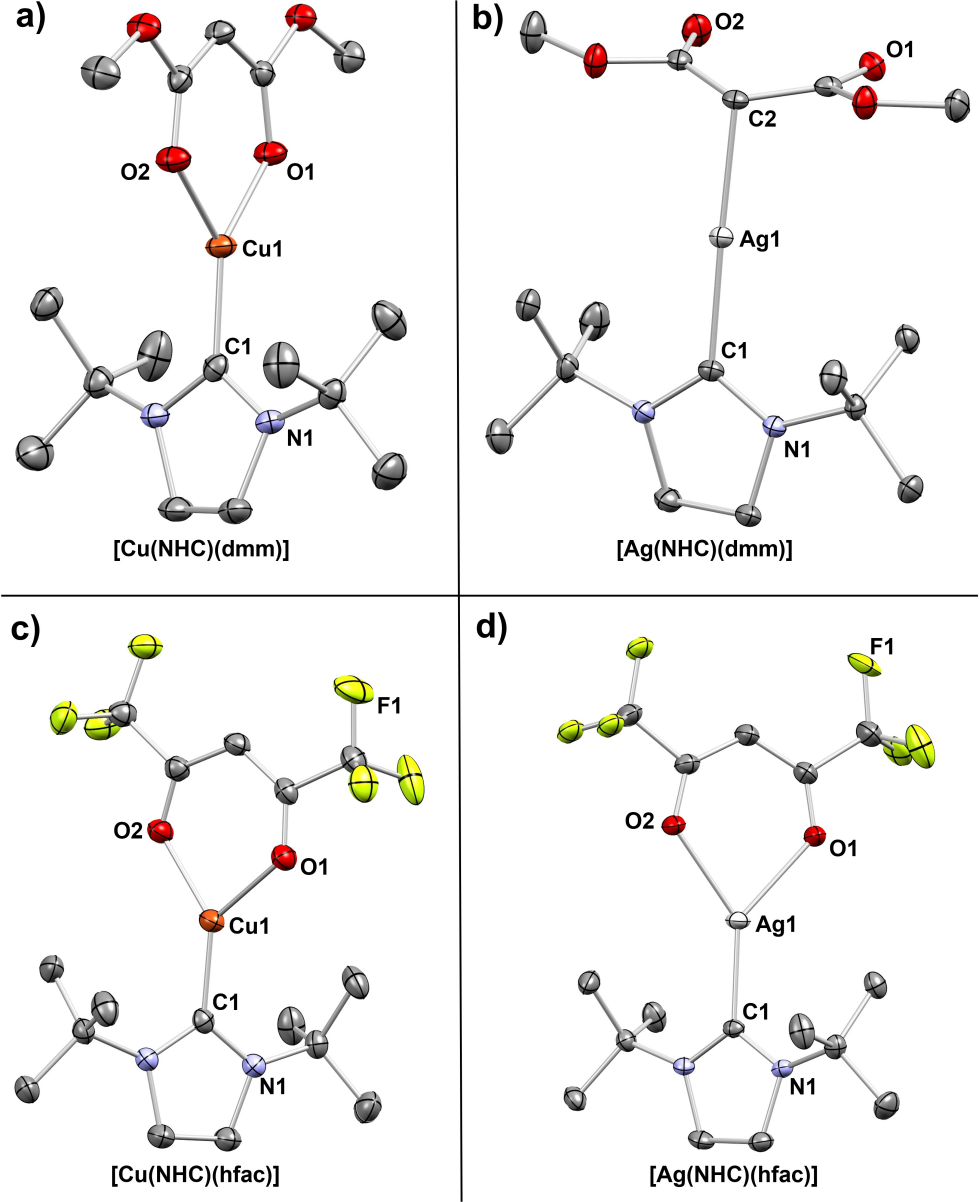
SC‐XRD structures for representative Cu and Ag complexes with the thermal ellipsoids shown at 50 % probability. Hydrogen atoms and disorders are omitted for clarity.

The low‐temperature solid‐state crystalline structures of the Cu and Ag complexes have been evaluated to gain a detailed understanding into their bonding and packing situation. A selected set of crystal structures is shown in Figure 2. All the complexes crystallize in a monomeric state, while no interactions between Cu−Cu or Ag−Ag are present within the crystal packing which indicates a strong steric shielding of the Cu and Ag nucleus. Nearly all complexes crystallize in a monoclinic crystal system with a space group of *P*2_1_/*c* or *P*2_1_/*n*, except for [Cu(^*t*Bu^NHC)(hfac)] and [Ag(^*t*Bu^NHC)(dmm)] which crystallize in a triclinic crystal system in the space group *P* $\bar{1}$ . This is the first indication that the packing and structure within the crystal is very similar for nearly all the complexes which is expected as the variation of the anionic diketonate backbone only introduces incremental changes to the overall crystalline structure of the complexes. Most interestingly, the structure of the complex [Ag(^*t*Bu^NHC)(dmm)] is very different from the general structural motif seen for all other complexes.

While all other complexes feature a bidentate coordination of the anionic diketonate ligand through M−O interactions and a monodentate M−C interaction of the carbene and the metal atom, [Ag(^*t*Bu^NHC)(dmm)] (Figure 2b) features a malonate ligand in which the endocyclic carbon atom is attached in a tetrahedral geometry to the Ag metal ion in a monodentate fashion. It should be noted that monomeric Ag complexes with carbanion‐Ag interactions are generally a rare occurrence in literature due to the inherently unstable nature of these compounds. This is easily reflected by the low stability of Ag alkyl compounds which are known to be only stable for a short period of time (seconds to minutes) at low temperatures, while the related Cu alkyls are even more unstable.^[34]^ In the present complex, a loss of resonance stabilization is apparent with C−O bond lengths in the range of 1.210 Å, which come close to the bond lengths of typical C=O double bonds in ketones and ketoesters.^[35]^ Moreover, the C−O bond in [Ag(^*t*Bu^NHC)(dmm)] is significantly shorter than for all other complexes (1.242 to 1.278 Å), where a resonance stabilization is further indicated by the formation of nearly planar Cu and Ag metallacycles. Additionally, IR measurements clearly indicate a strong diversion of the C−O stretching frequency (1718 cm^−1^) for [Ag(^*t*Bu^NHC)(dmm)] compared to the other complexes which is in the range of commonly known stretching frequencies for C=O double bonds (Figure S20 in the Supporting Information).^[36]^ The intermolecular interactions between the proton in the endocyclic bulk and the oxygen atoms of the carbonyl function (C=O⋅⋅⋅H−C) might stabilize this unusual bonding within the crystal packing as a layered structure along the *a*‐axis (illustrated in Figure S23). Even in solution the unusual structure seems to be retained as indicated by the chemical shift to the high field of the endocyclic proton in NMR as discussed earlier. The electron‐withdrawing nature of the C=O bonds located in β‐position to the carbanion and the electronic stabilization of the carbene might contribute to the overall stability of this complex.

**Figure 3:**
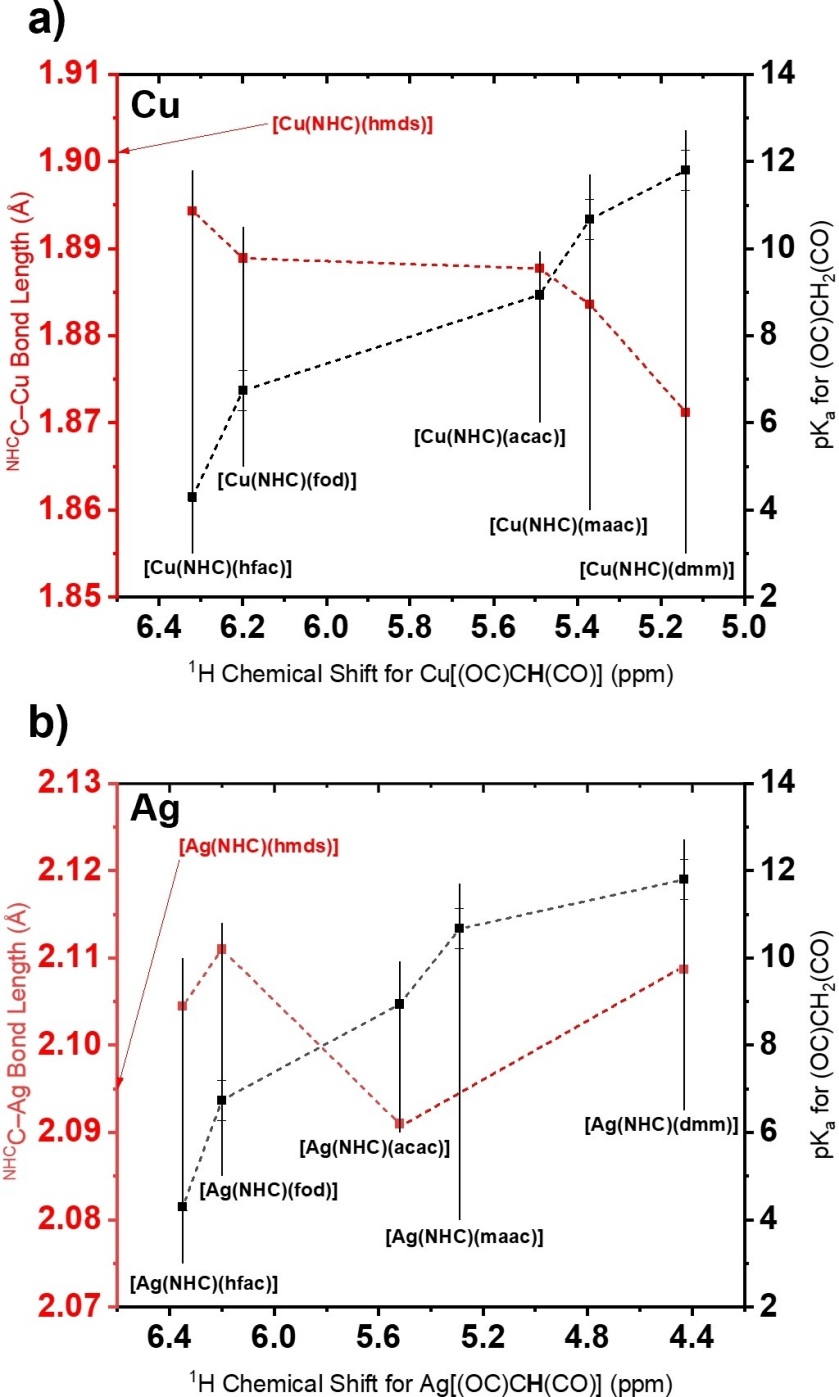
Depiction of the relation between the ^1^H NMR shifts of the proton within the endocyclic diketonate ligand, the p*K*_a_ values of the conjugated protonated diketonates (keto‐form) and carbene‐metal bond lengths for a) Cu and b) Ag complexes.

Small amounts of impurities were found as additional peaks in the ^1^H NMR of [Ag(^*t*Bu^NHC)(hfac)] and [Ag(^*t*Bu^NHC)(fod)] (Figures S8 and S9) that could not be clearly assigned initially. During the screening of suitable crystals for SC‐XRD measurements, it was apparent that a very small fraction in the form of sharp needles is present in the crystalline product mixture of both complexes. SC‐XRD measurements of these crystalline needles revealed an interesting structure (Figure S24) that represents a [Ag(hfac)_2_]_2_(NHC) dimeric cluster species with a closely related carbanion‐Ag interaction as already discussed for [Ag(^*t*Bu^NHC)(dmm)]. The reason for the formation might be a local overconcentration of the protonated hfac ligand in the reaction mixture during its addition to the starting material [Ag(NHC)(hmds)]. This might cause the formation of a dinuclear Ag species with two instead of one hfac ligands attached to the metal. The crystal structure of the crystalline residues, which were also observed for [Ag(^*t*Bu^NHC)(fod)], could not be determined due to poor crystal quality; however, it can be assumed that the structure might be analogous to the observed [Ag(hfac)_2_]_2_(NHC). For the Cu complexes, a correlation between the NMR shifts of the protons located at the endocyclic carbon atom within the diketonate ligand (−CH−) and the p*K*_a_ values of their conjugated acids in the keto‐form (−CH_2_−) can be considered. It clearly shows that the NMR signals are shifted downfield for lower p*K*_a_ values and thus a higher degree of electron‐withdrawing effect in the order hfac>fod>acac>maac>dmm can be assumed. The electron‐withdrawing effect of the coordinated diketonate should thus also influence the carbene‐metal interaction as less electron density is available at the metal nucleus for π‐electron backdonation to the carbenic carbon atom of the NHC which should elongate and thus weaken the C−M bond. A similar study was carried out by Bijou et al., in which the thermodynamic stability of heteroleptic Titanium‐diketonate complexes could be linked to the p*K*_a_ value of the diketonate species.^[37]^ This effect is observed for the Cu complexes (Figure 3a) with an elongation of the C−M bond from 1.871 (dmm) to 1.895 Å (hfac). Even though a relation between NMR shifts and p*K*_a_ values for the parent Ag complexes (Figure 3b) can be postulated as expected, there seems to be no significant influence of the electron‐withdrawal of the diketonate backbone on the actual C−M bond lengths which range from 2.091 (acac) to 2.111 Å (fod). The most significant difference can be allocated between [Ag(^*t*Bu^NHC)(acac)] and [Ag(^*t*Bu^NHC)(hfac)], where the C−M bond is elongated for [Ag(^*t*Bu^NHC)(hfac)]. Quite intriguingly is the fact that the already reported Cu complex [Cu(^*t*Bu^NHC)(hmds)] features an even longer C−M bond with 1.901 Å which might indicate an even lower electron density at the carbenic carbon atom or steric repulsion due to the larger trimethylsilyl groups in close spatial proximity to the *tert*‐butyl groups of the carbene.

**Figure 4:**
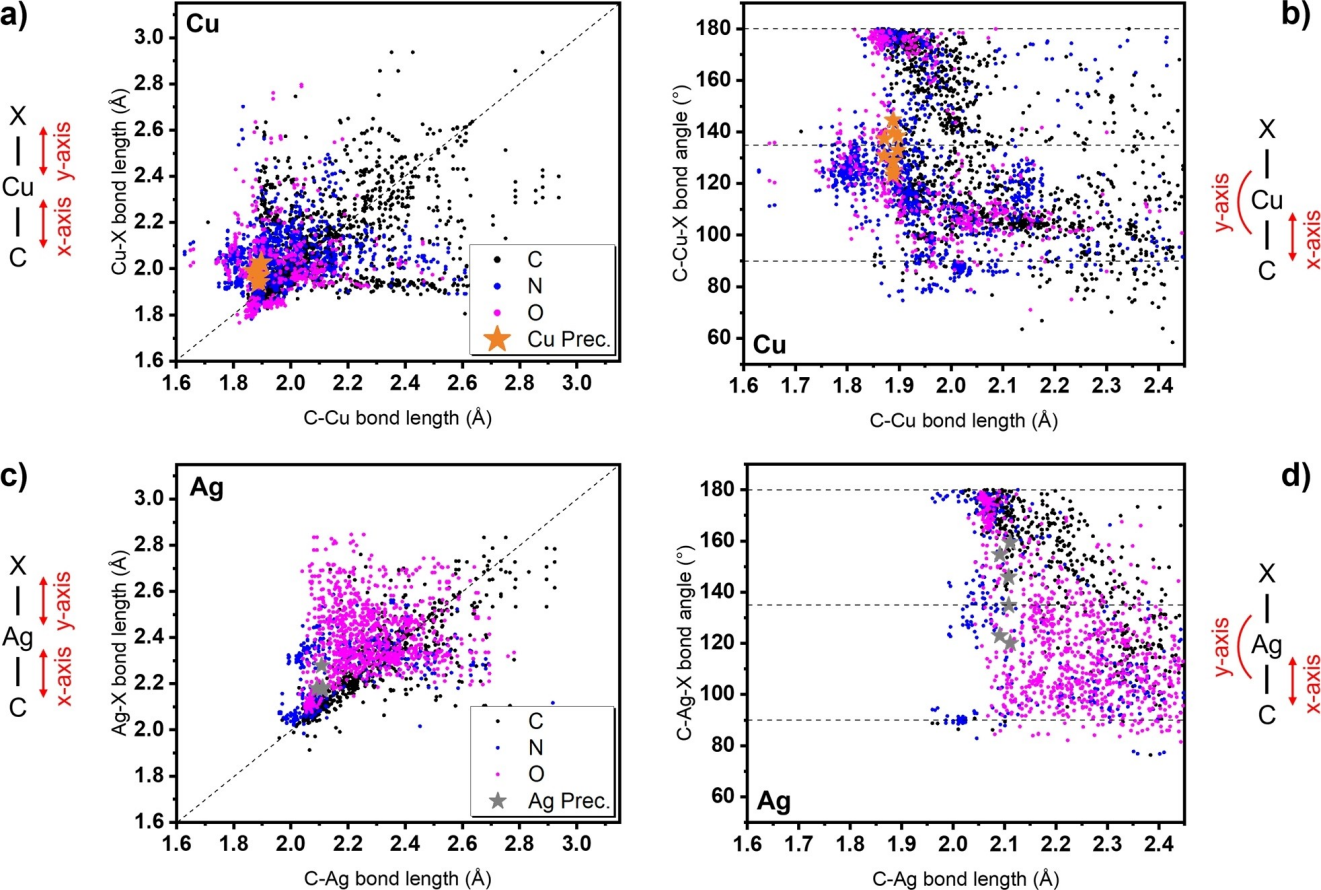
Data points from the CSD search plotted for the general bonding motif of C−M−X bonds. a) and c) The relation between bond lengths of C−M and M−X. b) and d) Display the relation between C−M bond lengths and C−M−O bond angles.

On the other hand, [Ag(^*t*Bu^NHC)(hmds)] with an C−M bond length of 2.095 Å does not follow a similar trend in this case.^[32]^ The O−Cu−O bite angles are sharper for the fluorinated complexes (hfac, fod) with 88° to 90° than for the non‐fluorinated ligands (e. g., acac) with 92°. While the Ag complexes follow this general trend, it should be noted that their O−Ag−O bond angles are significantly sharper (79.5° for [Ag(^*t*Bu^NHC)(hfac)] which might be explained by a higher mono‐cationic radius of Ag compared to Cu and causes the ligands to shift further away from the metal atom in the case of Ag.^[38]^ To study and compare the results obtained by SC‐XRD to a broader spectrum of similar complexes, we conducted a data search in the Cambridge Structural Database (CSD) with a general C−M−X (X=O, N, C; M=Cu, Ag) bonding motif for metalorganic Cu and Ag complexes.^[39]^ The results from the search are plotted in the graphs in Figure 4 and concentrate on C−M versus C−X bond lengths (graphs a and c) and C−M bond lengths versus C−X bond angles (graphs b and d). Datapoints for the bond lengths and angles from complexes obtained in this study are also included in the graphs and allow a comparability of the results to findings within the general published CSD literature. It is clearly apparent that the shortest observable C−M bond lengths between Cu and Ag complexes from this study are separated by around 0.2 Å which is very close to the difference in the mono‐cationic radius of Cu and Ag (0.17 Å).^[38]^ This is also reflected by a shift of most of the datapoints from similar Cu and Ag complexes at around 0.2 Å, irrespective of the second coordinated atom type X and was further confirmed in our previous studies.[^31^, ^32^] The complexes synthesized in this study feature C−M and C−O bonds that can be considered on the shorter end compared to literature known C−M−O bond lengths. Especially for Ag complexes, shorter and thus stronger bonds might be obtained for N‐coordinating anionic ligands indicated by a minimum bond length of 1.95 Å for the shortest M−N bond length reported in the CSD and thus should leave room for the optimization of the ligand architecture in the respective complexes. A comparison between the C−M−O bond angles in Cu and Ag complexes reveal that the coordination is preferentially linear (180°, e. g., [Cu(^*t*Bu^NHC)(hmds)]),[^30^, ^31^, ^33^] orthogonal (90°, e. g., [Cu(hmds)]_4_) or in‐between (135°, multidentate or bridged complexes).^[40]^ For Cu, the bond angles for complexes in this study and complexes found in the literature with Cu−O bonds at short C−Cu bond distances below 1.9 Å are highly localized around 180° and 135°, whereas for the parent Ag complexes the bond angles are more distorted and diffuse especially for C−Ag bond distances greater than 2.1 Å. This observation is furthermore validated by our results which indicate more diffuse C−M−O bond angles in the case of Ag. Interestingly, N‐coordinating ligands with C−Ag bond lengths below 2.1 Å feature a strong localization of bond angles at 180°, 90° and 135°. This highlights that in terms of bond lengths, bond angles, and thus also bond‐strengths, significant improvements might be achieved by a thoughtful choice of ligands that are most probably focused on N‐coordinating ligand systems with monodentate or multidentate bonding features, such as amidinate, diketiminate or stabilized amide ligands. Considerably shorter C−Ag (2.078 Å) and Ag−N (2.073 Å) bonds were obtained in a recent study by Arachchilage et al.^[41]^ with an monomeric NHC‐stabilized N‐coordinated Ag^I^ pyrazolate complex as potential precursor for ALD which is a first confirmation for the assumptions made in this section. We are currently working on complexes with a diverse set of anionic ligands to further confirm this general trend. Even though Cu and Ag complexes feature a remarkable similarity in their coordination chemistry, longer and thus weaker bonds strengths seem to be an inherent limitation for the ligand to Ag interaction and might render Ag complexes more unstable than their parent Cu complexes as further highlighted in the upcoming sections of this study.

### EI‐MS

**Figure 5:**
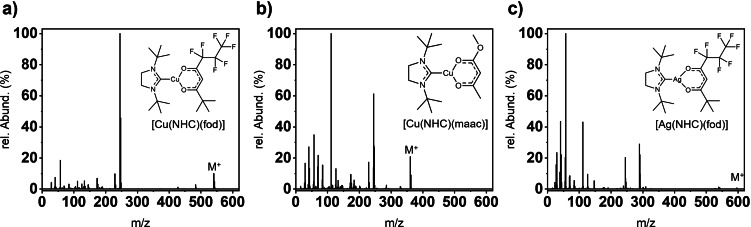
EI‐MS spectra with their fragmentation patterns highlighted for selected Cu and Ag complexes.

To envisage the fragmentation behavior and fragment stability in the gas‐phase after electron bombardment, EI‐MS was carried out. While all Cu complexes could be successfully evaluated by EI‐MS, only two Ag complexes, namely [Ag(^*t*Bu^NHC)(hfac)] and [Ag(^*t*Bu^NHC)(fod)] could be evaluated as the seemingly more unstable [Ag(^*t*Bu^NHC)(dmm)] and [Ag(^*t*Bu^NHC)(acac)] most probably decomposed before a good evaporation rate could be achieved in the EI‐MS vaporization chamber. All analyzable complexes feature a monomeric state in the gas phase under EI‐MS conditions as no significant peak was observed at higher *m*/*z* values than their respective M^+^ peak, which is visible for all complexes in their respective spectra (Figures 5 and S19, Table 1). Exemplarily, the obtained EI‐MS spectrum for the complex [Cu(^*t*Bu^NHC)(fod)] (Figure 5a) shows three significant and strong signals at *m*/*z* ratios of 540.35, 245.20 and 57.09 that could be assigned to the molecular mass of [Cu(^*t*Bu^NHC)(fod)] (M^+^, 10.2 %), a [Cu(NHC)]^.+^ fragment (100 %), and a *tert*‐butyl fragment (18.6 %), respectively. Only a small number of signals with a low intensity at lower *m*/*z* ratios could be observed, while a possible [Cu(fod)]^.+^ fragment could not be observed at all. A nearly identical fragmentation behavior is present for the closely related fluorinated complex [Cu(^*t*Bu^NHC)(hfac)] and also for [Cu(^*t*Bu^NHC)(acac)], although the number and intensity of smaller fragments is higher for the latter complex indicating a more pronounced degree of fragmentation to smaller molecular parts. For the complexes with ligands including ester functionality, namely [Cu(^*t*Bu^NHC)(maac)] (Figure 5b) and [Cu(^*t*Bu^NHC)(dmm)], the fragmentation towards a high degree of smaller molecular fragments is highly pronounced and is moreover indicated by a high rel. abundance for a [C_8_H_15_]^.+^ fragment at *m*/*z* 111.12 (100 %). This is expected for ligands with ester functionality, as they tend to show a high degree of fragmentation through the cleavage of the corresponding methoxy group and more pronounced chemical lability that intrinsically fragmentates according to McLafferty‐like rearrangements.^[42]^

**Table 1:** Selected fragments for the Cu and Ag complexes obtained from the EI‐MS data.

Compounds	*m*/*z* (rel. abundance)			
	[M(NHC)(X)]^.+^	[M(NHC)]^.+^	[C_8_H_15_]^.+^	[*t*Bu]^.+^
[Cu(NHC)(acac)]	344.05 (30 %)	**245.20 (100 %)**	111.12 (48 %)	57.09 (30 %)
[Cu(NHC)(dmm)]	376.33 (2 %)	245.20 (14 %)	**111.12 (100 %)**	57.09 (17 %)
[Cu(NHC)(maac)]	360.19 (21 %)	245.20 (62 %)	**111.12 (100 %)**	57.09 (35 %)
[Cu(NHC)(fod)]	540.35 (10 %)	**245.20 (100 %)**	111.12 (5 %)	57.09 (19 %)
[Cu(NHC)(hfac)]	452.12 (25 %)	**245.20 (100 %)**	111.12 (7 %)	57.09 (16 %)
[Ag(NHC)(fod)]	596.36 (1 %)	289.18 (29 %)	111.12 (43 %)	**57.09 (100 %)**
[Ag(NHC)(hfac)]	451.55 (6 %)	**289.18 (100 %)**	111.12 (39 %)	57.09 (39 %)

The Ag complex [Ag(^*t*Bu^NHC)(hfac)] showed a comparable fragmentation behavior to [Cu(^*t*Bu^NHC)(hfac)] and [Cu(^*t*Bu^NHC)(fod)], but still a higher degree of fragmentation to smaller fragments indicated by a higher intensity of peaks at lower *m*/*z* values could be denoted. Contrasting this result, [Ag(^*t*Bu^NHC)(fod)] (Figure 5c) presents a strong fragmentation with a high relative abundance for the [*t*Bu]^.+^ fragment at *m*/*z* 57.09 (100 %) and a very low intensity of the M^+^ peak with only 0.65 %. This confirms that the Ag complexes (with one exception) seem to be highly unstable after ionization and possess a higher susceptibility towards fragmentation compared to their parent Cu complexes. Notably, the fragmentation behavior is somewhat different to the EI‐MS fragmentation observed for [Ag(NHC)(hmds)] reported in our earlier work, as in this case a Ag fragment with an anionic amide backbone [Ag(hmds)]^.+^ was clearly visible in the spectra and a comparable fragment is not observable for any of the diketonate complexes characterized in this study.

It can be concluded that fluorinated ligands, which withdraw electron density from the Cu or Ag nucleus causes shorter and most probably stronger bonds of the NHC to the metal as discussed earlier, showing a higher overall stability under EI‐MS conditions. This is especially apparent for the Ag complexes, for which only [Ag(^*t*Bu^NHC)(hfac)] shows a comparably high resistance towards strong fragmentation. In general, the Cu complexes seem to be more stable and less prone to strong fragmentation during EI‐MS compared to the Ag complexes. The EI‐MS experiments should give a first hint on the molecular and fragment stabilities under these conditions and should enable to understand the influence of the anionic ligand on complex stabilities which might help to gain a better understanding of the thermal evaporation behavior of the complexes which is discussed in the next section.

### Thermal and reactivity assessment

**Figure 6:**
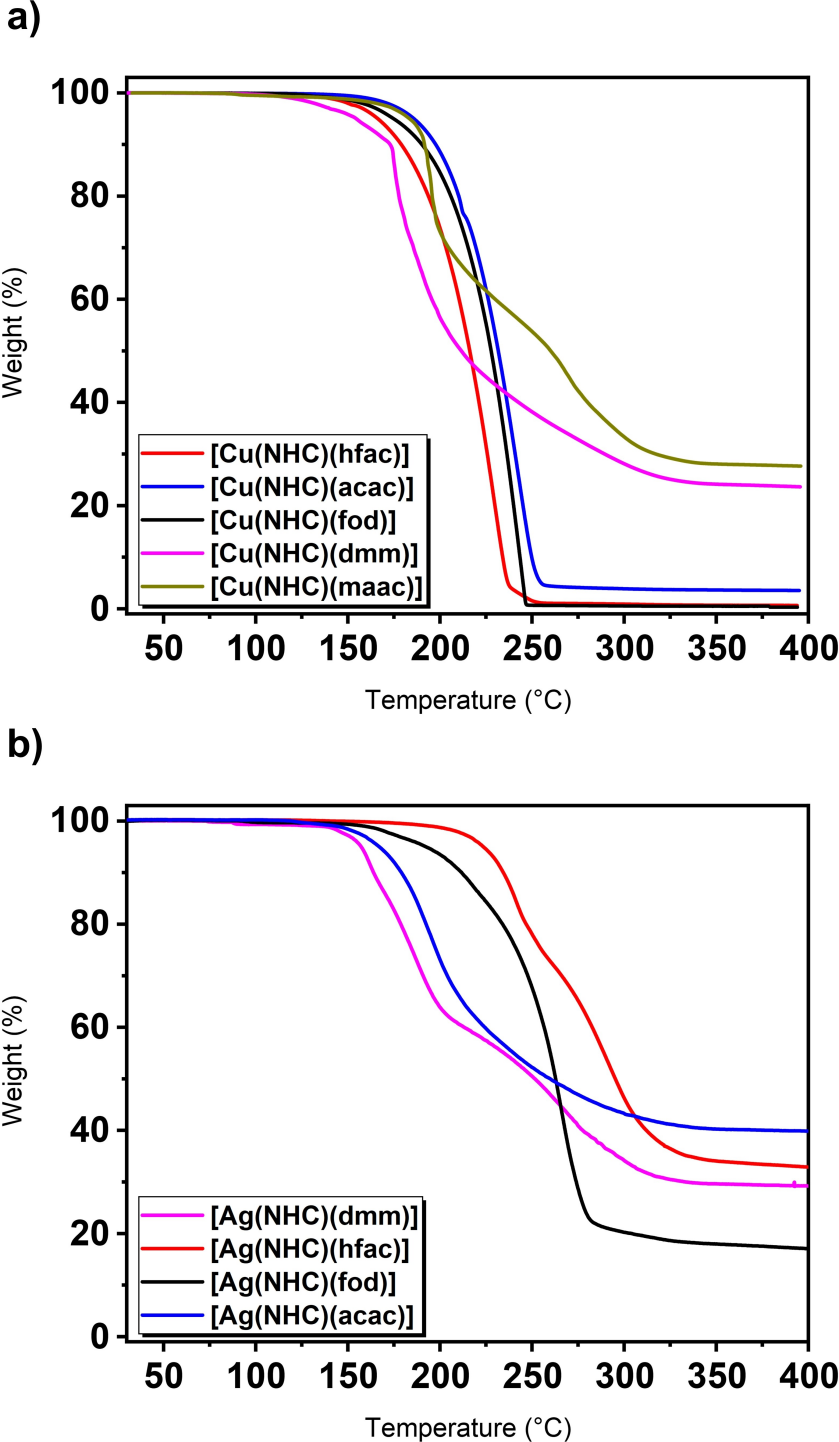
Graphs for the TGA of all a) Cu and b) Ag complexes which display the distinct evaporation profiles by heating from RT to 400 °C.

The assessment of thermal properties is one of the most crucial factors for a successful application of these targeted complexes as precursors in actual vapor phase deposition processes. For this, thermogravimetric analysis (TGA) can determine the evaporation profile of the employed compound and was carried out for all synthesized Cu and Ag complexes in this study (Figure 6, Table 2). The Cu complexes feature a strongly varying evaporation profile in the TGA experiments: [Cu(^*t*Bu^NHC)(hfac)], [Cu(^*t*Bu^NHC)(fod)] and [Cu(^*t*Bu^NHC)(acac)] feature a clean single‐step volatilization curve resulting in residual weights of below 3 %.

**Table 2:** Overview of thermal parameters for the different Cu and Ag precursors obtained by TGA.

Compound	*T*_o_ [°C]	*T*_m_ [°C]	*T*_d_ [°C]	*W*_r_ [°C]	*T* (1 Torr) [°C]
[Cu(NHC)(fod)]	143.0	130	–	0.5	165
[Cu(NHC)(hfac)]	138.8	152	–	0.6	155
[Cu(NHC)(acac)]	160.7	211	–	3.6	173
[Cu(NHC)(maac)]	143.0	dec.	–	27.7	–
[Cu(NHC)(dmm)]	118.8	dec.	–	23.5	–
[Ag(NHC)(fod)]	158.5	98	–	17.0	–
[Ag(NHC)(hfac)]	194.5	116	230	32.9	–
[Ag(NHC)(acac)]	144.2	dec.	142	40.1	–
[Ag(NHC)(dmm)]	135.4	dec.	113	29.3	–

With an onset of evaporation (after 1 % weight loss) at 138.8 °C, [Cu(^*t*Bu^NHC)(hfac)] is the most volatile compound in this row followed closely by [Cu(^*t*Bu^NHC)(fod)] at 143.0 °C. Evaporation of both compounds results in low residual weights of 0.5 and 0.6 % after heating to 550 °C, respectively. The least volatile compound in this row is [Cu(^*t*Bu^NHC)(acac)] which features an onset of volatilization at 160.7 °C with a residual weight of 3.6 %. Among the mostly overlapping evaporation windows it can be assumed that [Cu(^*t*Bu^NHC)(hfac)] and [Cu(^*t*Bu^NHC)(fod)] feature the highest thermal stability which is indicated by lower residual weights compared to [Cu(^*t*Bu^NHC)(acac)].

The trends seen in the onset of evaporation points as an indication for their volatilities could be confirmed by determining the vapor pressure of the three complexes by stepped‐isothermal‐thermogravimetry and calculation of the corresponding vapor pressures according to the Langmuir equation using a route introduced by Kunte et al.^[43]^ (Figure S33). [Cu(^*t*Bu^NHC)(hfac)] features the lowest temperature (155 °C) where 1 Torr of vapor pressure is reached and thus has the highest volatility among the other complexes, closely followed by [Cu(^*t*Bu^NHC)(fod)] (165 °C) and [Cu(^*t*Bu^NHC)(acac)] (173 °C). The vapor pressure of [Cu(^*t*Bu^NHC)(acac)] is closely comparable to that of [Cu(^*t*Bu^NHC)(hmds)] for which Coyle et al.^[33]^ determined the same temperature (173 °C) for reaching a vapor pressure of 1 Torr. Remarkably, [Cu(^*t*Bu^NHC)(acac)] has the lowest molecular weight among the three complexes, yet still the lowest volatility which can mostly be attributed to a lower degree of attractive van‐der‐Waals interactions in [Cu(^*t*Bu^NHC)(fod)] and [Cu(^*t*Bu^NHC)(hfac)] due to the repulsive interactions of the fluorinated ligands. For the other two complexes [Cu(^*t*Bu^NHC)(maac)] and [Cu(^*t*Bu^NHC)(dmm)], only a poor thermal stability is apparent and indicated by a multiple‐step weight loss and higher residual weights of 27.7 and 23.5 %, respectively.

This further supports the findings by EI‐MS which showed a high degree of fragmentation and inherent instability of the latter complexes under these conditions and further match the findings of our former study on Hf‐ and Dy‐based malonate complexes.[^44^, ^45^] On the contrary, most of the Ag complexes feature a multiple‐step evaporation behavior while only [Ag(^*t*Bu^NHC)(fod)] evaporates in a single step with an onset of 158 °C but seemingly decomposes slightly during evaporation yielding a residual weight of 17.0 %. All the other Ag complexes show inherently thermally unstable properties which led to residual weights of 30–40 %. The relatively high thermal stability for the complex [Ag(^*t*Bu^NHC)(fod)] is also apparent when evaluating the differential scanning calorimetry (DSC) curves of the parent silver complexes (Figure S35–S38): A sharp exothermic peak in the DSC curve which might indicate a decomposition event is not clearly visible for [Ag(^*t*Bu^NHC)(fod)]. Thus, it can be expected that [Ag(^*t*Bu^NHC)(fod)] evaporates with a minor component of decomposition at higher temperatures which leads to a residual weight of 17.0 %. All the other complexes feature sharp exothermic peaks in their respective DSC curves before a significant amount of evaporation takes place which naturally lead to high residual weights >30 %. Overall, the thermal stability among the employed diketonate ligands according to their exothermic decomposition events in DSC (Table 2) for the Ag complexes can be ranked as fod>hfac>acac>dmm, while [Ag(^*t*Bu^NHC)(maac)] was even too unstable to be properly isolated during synthesis. Interestingly, [Ag(^*t*Bu^NHC)(fod)] shares very similar thermal properties (Figure S39) to the already established Ag precursor [Ag(^*t*Bu^NHC)(hmds)] which was used previously by our group in the spatial ALD of silver metal. This at least indicates, that [Ag(^*t*Bu^NHC)(fod)] might be used in ALD experiments in future studies to explore the behavior of this complex under actual ALD conditions. All the other synthesized complexes might not be specifically suited for the ALD of silver metal but still feature highly interesting bonding behavior and chemistry. These results further highlight that the simple transfer of highly stable ligand systems from Cu to Ag do not automatically ensures a volatile and thermally stable precursor for vapor deposition processes such as ALD. As a final assessment, the reactivity of all compounds was tested in solution and monitored visually by NMR experiments (Figures S25–S33). For this, a commonly used reducing agent in ALD,^[46]^ namely dimethylaminoborane adduct (BH_3_ ⋅ NHMe_2_), was added to the respective complexes (ca. 30 mg) dissolved in deuterated benzene in a slight excess. Directly after addition, a shiny metallic‐looking Cu layer was forming on the sides of the NMR tubes for the Cu complexes, while an off‐white with a matt finish precipitate formed after addition of the borane to the Ag complexes. The progress of the reaction was monitored by NMR directly after the reaction was initiated and a complete conversion of the complexes to various products could be evaluated from the respective spectra. Even though a complete conversion of the complexes could be monitored by NMR, no products from the reaction such as organic by‐products could be isolated from the mixture afterwards that would reveal possible reaction mechanisms for the reduction of the complexes to Cu and Ag metal in solution. As a first test, this at least indicates the high reactivity of the complexes towards potential reducing agents which might be used in ALD experiments at a later stage for the formation of Cu metal thin films. Among the complexes synthesized, [Cu(^*t*Bu^NHC)(acac)] features the most promising properties to be used in ALD processes, as it is thermally stable, volatile and reactive. Even though [Cu(^*t*Bu^NHC)(fod)] and [Cu(^*t*Bu^NHC)(hfac)] feature better thermal characteristics, for example, higher volatility and thermal stability, the fluorinated side chains might lead to incorporation of unwanted fluorine residues in the thin films. Thus, we employed [Cu(^*t*Bu^NHC)(acac)] in proof‐of‐principle depositions using hydroquinone as the co‐reactant to unveil its potential in ALD experiments, which is discussed in the next section.

### Deposition experiments

**Figure 7:**
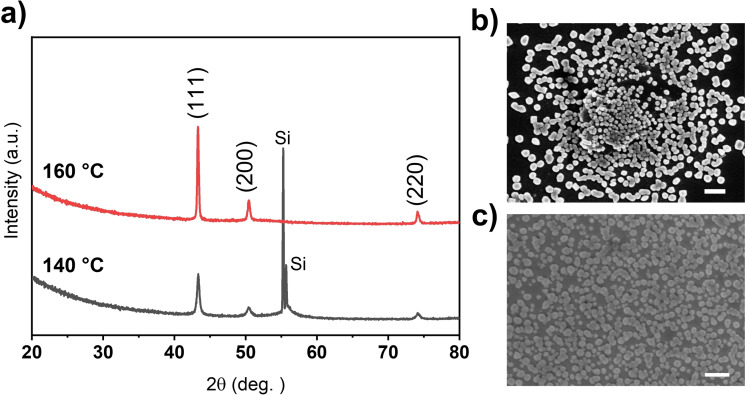
Structural characterization data for nanoparticulate Cu deposited on Si from [Cu(^*t*Bu^NHC)(acac)] and HQ: a) GIXRD patterns. Top‐view SEM images for deposits processed at b) 160 and c) 145 °C; scale bars: 300 nm.

Preliminary attempts were pursued to evaluate [Cu(^*t*Bu^NHC)(acac)] as an ALD precursor for the deposition of Cu on Si(100) substrates by using hydroquinone (HQ) as the reductant. In our previous studies,[^47^, ^48^] we had demonstrated the capability of organic HQ to reduce Cu^2+^ to Cu^0^; where [Cu(acac)_2_] was used as the Cu precursor. Herein, we show for the first time that a similar process works also with a Cu^I^ precursor. For these deposition experiments, we fixed the precursor sublimation temperatures as follows: 115 °C for Cu(^*t*Bu^NHC)(acac) and 90 °C for HQ. The precursor pulsing sequence was: 4‐s Cu(^*t*Bu^NHC)(acac)/8‐s N_2_/4‐s HQ/8‐s N_2_. The deposition temperature was optimized starting from 120 °C, and it was observed that highly crystalline nanoparticulate Cu deposits were successfully grown in the temperature range of 145 to 160 °C. The grazing incidence X‐ray diﬀraction (GIXRD) patterns shown in Figure 7a reveal that the obtained nanoparticulate deposits are polycrystalline, and also that they are free from any crystalline foreign inclusions, especially of Cu oxides. This is a remarkable result, as it shows that using [Cu(^*t*Bu^NHC)(acac)] as the precursor, metallic nanoparticulate Cu deposits can be grown at appreciably low temperatures. Tentatively, we attribute this to the monovalent Cu in [Cu(^*t*Bu^NHC)(acac)], instead of the divalent Cu in the most commonly employed Cu precursors.

By increasing the deposition temperature, the crystallinity was further enhanced, as indicated with the intense GIXRD reflections for the nanoparticulate deposits formed at 160 °C. However, the ALD process was found to deviate from the normal growth pattern beyond 160 °C, resulting in strong nanoparticle island formation. From the SEM images shown in Figure 7b and c, a certain level of island type growth is seen even for the nanoparticles deposited at 160 °C, whereas the agglomerates deposited at 145 °C show more connectivity among the crystallites; however, the nanoparticulate character of the deposit is still apparent. A detailed study on copper metal island coalescence in ALD can be found in an article by Hagen et al.^[49]^ On the other hand, Rutherford backscattering spectrometry (RBS) and nuclear reaction analysis (NRA) indicated a good purity for the deposits formed at the higher temperatures, as the carbon content decreased from 15.0 at.% for the deposits formed at 145 °C to 8.8 at.% for that deposited at 165 °C. More details with regards to the RBS and NRA measurements can be found in Table S4 and Figures S40 and S41. These results underline the need of further process optimization and in‐depth characterization of the obtained deposits, including X‐ray photoelectron spectroscopy (XPS) and resistivity measurements, which we will undertake in our future studies.

## Conclusion

Five new monomeric Cu complexes and four new closely related Ag complexes with the general formula [M(^*t*Bu^NHC)(R)] have been successfully synthesized in this study. Through a rational and incremental change of the substitution pattern within the anionic backbone based on diketonates, clear trends for their structural and thermal parameters could be observed depending on the employed anionic backbone. NMR spectroscopy and single‐crystal X‐ray diffraction (SC‐XRD) revealed a first interesting trend in the bonding and structure of the complexes: although all the complexes are monomeric in the solid and liquid phases, their structure, bond lengths and bond angles not only strongly differ between complexes with different anionic backbones, but also deviate strongly for the two employed metals. The structural trends and influences seen for both metals could be verified and compared to metalorganic complexes of a similar nature obtained from an extensive search in the Cambridge Structural Database; this further highlighted the similarities and differences for complexes featuring either Cu or Ag as the central metal atom. A first insight into the stability of the complexes could be obtained by EI‐MS, which revealed monomeric nature in the gas phase for the analyzed complexes and a more pronounced fragmentation behavior for the Ag complexes in comparison to the directly related Cu complexes. The evaporation profile and thermal characteristics revealed that the Cu complexes have a higher thermal stability than the parent Ag complexes. Vapor pressure measurements for selected Cu precursors were performed, and [Cu(^*t*Bu^NHC)(hfac)] was shown to have the highest vapor pressure of all complexes. NMR studies of the reactivity of all complexes towards a borane‐based reducing agent in solution revealed their high reactivity for the formation of Cu or Ag metal. As a proof‐of‐concept, the feasibility of Cu precursor [Cu(^*t*Bu^NHC)(acac)] in ALD was demonstrated with a low‐temperature (145 °C) deposition of metallic Cu by using hydroquinone as the reducing agent. Analysis by XRD, SEM and RBS/NRA of the resulting deposits confirmed the successful deposition of Cu nanoparticulate agglomerates and highlighted the applicability of this precursor class in vapor‐phase deposition processes. The results obtained in this study set a new milestone in understanding the influence of systematic anionic ligand choice on the applicability of Cu^I^ and Ag^I^ precursors in vapor‐phase deposition processes and will further help to develop new precursors for Cu‐ and Ag‐containing thin and nanoparticulate films in the future.

## Experimental Section

The synthesis and handling of all reagents and products was carried out using standard *Schlenk* protocols with Ar as an inert gas to prevent contact with ambient air and moisture. The products of all reactions were stored and handled inside a dual MBraun 300 Glovebox system. The solvents were dried by an MBraun solvent purification system (SPS) and stored under inert gas atmosphere over molecular sieves (4 Å). All commercially available reagents were used without further purification. NMR measurements were performed on a Bruker Avance III 400, Avance III 300 and DPX‐200 spectrometers in NMR tubes under inert atmosphere with degassed and dried deuterated solvents. EA measurements (CHNS) were performed on a Vario Micro Cube from Elementar Analysensysteme GmbH and the samples were prepared in sealed tin crucibles inside a glovebox. Thermogravimetric analysis (TGA) was carried out with a Seiko Exstar TG/DTA 6500SII by employing a constant nitrogen flow (300 mL min^−1^) and a constant heating rate (5 K min^−1^) for the only mildly air‐sensitive Cu compounds. The TGAs for the highly sensitive Ag complexes were carried out in a Netzsch STA 409 PC LUXX which was operated in an argon filled glovebox. A constant nitrogen flow (90 sccm) and a constant heating rate (5 K min^−1^) was employed. For each measurement and on both devices, approx. 10 mg of the respective compound was used. Single crystals of the respective Cu and Ag compounds were crystallized from concentrated solutions in hexane, pentane, tetrahydrofuran or diethyl ether at −35 °C. A suitable crystal was selected under a microscope in perfluoro‐ether oil and mounted inside a flexible loop on a SuperNova (Atlas) diffractometer. The crystals were cooled to 100 K during data collection. Using Olex2,^[50]^ the structure was solved with the SHELXT structure solution program using Intrinsic Phasing and refined with the SHELXL refinement package using Least Squares minimization.[^51^, ^52^] Deposition Numbers 2104335 (for [Ag(NHC)(fod)]), 2104335 (for [Cu(NHC)(dmm)]), 2104337 (for [Cu(NHC)(fod)]), 2104338 (for [Ag(NHC)(hfac)]), 2104339 (for [Cu(NHC)(maac)]), 2104340 (for [Ag(NHC)(dmm)]), 2104341 (for [Cu(NHC)(acac)]), 2104342 (for [Ag(NHC)(acac)]), 2104343 (for [Ag(hfac)_2_]_2_(NHC)), 2104344 (for [Cu(NHC)(hfac)]) contain the supplementary crystallographic data for this paper. These data are provided free of charge by the joint Cambridge Crystallographic Data Centre and Fachinformationszentrum Karlsruhe Access Structures service.

The p*K*_a_ values were obtained as predicted properties from the service “SciFinder; Chemical Abstracts Service: Columbus, OH.”

All the thin films under discussion here were deposited on 2×2 cm^2^ silicon substrate in a commercial flow‐type hot‐wall ALD reactor (F‐120 by ASM Ltd). Both [Cu(^*t*Bu^NHC)(acac)] and hydroquinone (HQ; benzene‐1,4‐diol) precursors were placed inside the reactor in open boats. Nitrogen (N_2_, 99.999 %, flow rate at 300 SCCM, Schmidlin UHPN 3000 N_2_ generator) was used as both the carrier and purge gas for the deposition process. The reactor pressure was around 3 to 5 mbar.

Grazing incidence X‐ray diﬀraction (GIXRD; X'Pert MPD PRO Alfa 1, PANalytical; Cu−Kα radiation; incident angle 0.5°) was used for investigating the crystallinity of the films. The surface morphology of the deposited thin films was studied using scanning electron microscope (SEM, Hitachi S‐4700). For the SEM analysis, sample specimen was mounted on carbon tape and the analysis was performed at a voltage of 10 kV and current of 15 μA.

Rutherford backscattering spectrometry (RBS) and nuclear reaction analysis (NRA) were performed at the RUBION facility (Ruhr University Bochum). In the RBS experiments, a beam (2.0 MeV 4He^+^ ions, intensity 20–40 nA) penetrated the whole film at an angle of 7°. Scattered particles were observed by a solid‐state detector which was placed at an angle of 160°. NRA experiments were conducted to obtain information on the atomic density of lighter elements such as carbon, nitrogen or oxygen. A beam of deuterons (1.0 MeV) penetrated the whole film, and the emitted protons were recorded at an angle of 135°. To shield the detector from scattered deuterons, a 6 μm Nickel foil was used. To systematically evaluate the obtained RBS and NRA spectra, the software SIMNRA was used.^[53]^

The starting materials [Cu(NHC)(hmds)] and [Ag(NHC)(hmds)] which are used in all following reactions for the formation of the final complexes was synthesized and characterized using a procedure reported earlier by our group (Boysen et al.).[^31^, ^32^]

*[Cu(NHC)(acac)]*: The starting material [Cu(NHC)(hmds)] (4.2 g, 10.3 mmol) is dissolved in 100 mL of hexane and acetylacetone (1.0 g, 10.3 mmol) is slowly added to the solution at RT and stirred. The resulting microcrystalline yellow precipitate is allowed to settle at the bottom of the flask after which the solvent is decanted. After washing the solid with hexane, it was dried under vacuum. Yield: 3.2 g (89 %). ^1^H NMR (300 MHz, C_6_D_6_): *δ*=5.47 (s, O=C−C*H*−C=O), 2.75 (s, N−C*H_2_*−C*H_2_*−N), 2.05 (s, O=C−C*H*_3_), 1.41 ppm (s, NC(C*H*_3_)_3_). ^13^C NMR (75 MHz, C_6_D_6_): *δ*=203.2 (s, N−*C*−N), 189.5 (s, O=C−*C*H−C=O), 99.0 (s, O−*C*−CH−*C*−O), 54.8 (s, O=C−*C*H_3_), 45.2 (s, N−*C*H_2_−*C*H_2_−N), 30.2 (s, N*C*(CH_3_)_3_), 28.7 ppm (s, NC(*C*H_3_)_3_). Elem. anal. calcd (%): N 8.12, C 55.71, H 8.47; found: N 8.56, C 55.28, H 8.51. FTIR: $\tilde{v}$ (cm^−1^)=2960 (s, CH_3_), 1597 (s, C=O).

*[Cu(NHC)(fod)]*: The starting material [Cu(NHC)(hmds)] (5 g, 12.3 mmol) is dissolved in 100 mL of hexane and 2,2‐dimethyl‐6,6,7,7,8,8,8‐heptafluorooctane‐3,5‐dione (3.65 g, 12.3 mmol) is slowly added to the solution at RT and stirred. The resulting orange suspension is mildly heated to completely dissolve the solid and stored at −35 °C to afford orange crystals which are dried under vacuum. Yield: 5.8 g (87 %). ^1^H NMR (400 MHz, C_6_D_6_): *δ*=6.21 (s, O=C−C*H*−C=O), 2.70 (s, N−C*H_2_*−C*H_2_*−N), 1.30 (s, O=CC(C*H*_3_)_3_), 1.18 ppm (s, NC(C*H*_3_)_3_). ^13^C NMR (101 MHz, C_6_D_6_): *δ*=204.96 (s, N‐*C*‐N or O−*C*C(CH_3_)_3_), 201.8 (s, N−*C*−N or O−*C*C(CH_3_)_3_), 172.3 to 171.9 (m, O−*C*−CF_2_−CF_2_−CF_3_), 120.8 to 120.1 (m, −*C*F_2_−CF_2_−CF_3_), 117.9 to 117.2 (m, −CF_2_−CF_2_−*C*F_3_), 111.8 to 111.3 (m, −CF_2_−*C*F_2_−CF_3_), 91.0 (s, O=C−*C*H−C=O), 54.7 (s, N−*C*H_2_−*C*H_2_−N), 45.2 (s, N*C*(CH_3_)_3_), 42.2 (s, O=C*C*(CH_3_)_3_)), 29.9 (s, (NC(*C*H_3_)_3_)), 28.1 ppm (s, (CC(*C*H_3_)_3_)). Elem. anal. calcd (%): N 5.18, C 46.62, H 5.96; found: N 5.33, C 45.26, H 5.64. FTIR: $\tilde{v}$ (cm^−1^)=2960 (s, CH_3_), 1622 (s, C=O).

*[Cu(NHC)(dmm)]*: The starting material [Cu(NHC)(hmds)] (0.5 g, 1.23 mmol) is dissolved in 10 mL of pentane and dimethyl propanedioate (0.16 g, 1.23 mmol) is slowly added to the solution and stirred. To the pale‐yellow suspension, 20 mL of pentane is added and mildly heated to completely dissolve the solid. Storing the solution at −35 °C overnight affords colorless crystals which are dried under vacuum. Yield: 0.37 g (71 %). ^1^H NMR (200 MHz, C_6_D_6_): *δ*=5.11 (s, O=C−C*H*−C=O), 3.64 (s, N−C*H_2_*−C*H_2_*−N), 2.70 (s, O=C−O−CH_3_), 1.39 ppm (s, NC(C*H*_3_)_3_). ^13^C NMR (50 MHz, C_6_D_6_): *δ*=175.4 (s, O=*C*−CH−*C<C*=>O), 64.8 (s, O=C−*C*H−C=O), 54.8 (s, O=C−O−*C*H_3_), 50.0 (s, N−*C*H_2_−*C*H_2_−N), 45.2 (s, N*C*(CH_3_)_3_), 30.1 ppm (s, NC(*C*H_3_)_3_), N−*C*−N not observed. Elem. anal. calcd (%): N 7.43, C 50.98, H 7.75; found: N 8.38, C 50.54, H 7.68. FTIR: $\tilde{v}$ (cm^−1^)=2960 (s, CH_3_), 1651 (s, C=O).

*[Cu(NHC)(maac)]*: The starting material [Cu(NHC)(hmds)] (0.5 g, 1.23 mmol) is dissolved in 10 mL of hexane and methyl acetoacetate (0.14 g, 1.23 mmol) is slowly added to the solution at RT and stirred for 72 h. To the grey suspension 10 mL of pentane is added and mildly heated to completely dissolve the solid. Storing the solution at −35 °C overnight affords colorless crystals which are dried under vacuum. Yield: 0.15 g (33 %). ^1^H NMR (200 MHz, C_6_D_6_): *δ*=5.34 (s, O=C−C*H*−C=O), 3.59 (s, N−C*H_2_*−C*H_2_*−N), 2.74 (s, O=C−CH_3_), 2.11 (s, O=C−O−CH_3_), 1.40 ppm (s, NC(C*H*_3_)_3_). ^13^C NMR (50 MHz, C_6_D_6_): *δ*=202.6 (s, N‐*C*‐N), 188.2 (s, O=*C*−O−CH_3_), 172.9 (s, O=*C*−CH_3_), 83.4 (s, O=C−*C*H−C=O), 54.8 (s, N−*C*H_2_−*C*H_2_−N), 49.6 (s, O=C−O−*C*H_3_), 45.2 (s, N*C*(CH_3_)_3_), 30.1 (s, NC(*C*H_3_)_3_), 28.4 ppm (s, C*C*H_3_). Elem. anal. calcd (%): N 7.76, C 53.24, H 8.10; found: N 8.30, C 53.07, H 8.11. FTIR: $\tilde{v}$ (cm^−1^)=2960 (s, CH_3_), 1634 (s, C=O).

*[Cu(NHC)(hfac)]*: The starting material [Cu(NHC)(hmds)] (0.5 g, 1.23 mmol) is dissolved in 10 mL of hexane and 1,1,1,5,5,5‐hexafluoropentane‐2,4‐dione (0.26 g, 1.23 mmol) is slowly added to the solution at RT which is stirred for 2 h. Bright red crystals formed in the solution which are separated from the solvent and dried under vacuum. Yield: 0.4 g (71 %). ^1^H NMR (400 MHz, C_6_D_6_): *δ*=6.30 (s, O=C−C*H*−C=O), 2.65 (s, N−C*H_2_*−C*H_2_*−N), 1.19 ppm (s, NC(C*H*_3_)_3_). ^13^C NMR (101 MHz, C_6_D_6_): *δ*=199.9 (s, N‐*C*‐N), 177.6 (s, O=*C*−CH−*C*=O), 88.9 (s, O=C−*C*H−C=O), 54.6 (s, N−*C*H_*2*_−*C*H_*2*_−N), 45.1 (s, N*C*(CH_3_)_3_), 29.9 ppm (s, NC(*C*H_3_)_3_). Elem. anal. calcd (%): N 6.19, C 42.43, H 5.12; found: N 6.22, C 42.07, H 4.94. FTIR: $\tilde{v}$ (cm^−1^)=2960 (s, CH_3_), 1644 (s, C=O).

*[Ag(NHC)(acac)]*: The starting material [Ag(NHC)(hmds)] (0.3 g, 0.66 mmol) is dissolved in 5 mL of diethyl ether and acetylacetone (0.066 g, 0.66 mmol) is added to the solution at RT and stirred. The solvent was removed under vacuum and exchanged by 10 mL THF after which the resulting clear solution was concentrated and stored at −35 °C overnight to afford colorless crystals. Yield: 0.045 g (17 %). ^1^H NMR (200 MHz, C_6_D_6_): *δ*=5.51 (s, O=C−C*H*−C=O), 2.66 (s, N−C*H_2_*−C*H_2_*−N), 2.16 (s, O=C−C*H*_3_), 1.27 ppm (s, NC(C*H*_3_)_3_). ^13^C NMR (50 MHz, C_6_D_6_): *δ*=190.4 (s, O=C−*C*H−C=O), 98.2 (s, O=*C*−CH−*C*=O), 54.9 (s, O=C−*C*H_3_), 45.4 (s, N−*C*H_2_−*C*H_2_−N), 30.5 (s, N*C*(CH_3_)_3_), 29.7 ppm (s, NC(*C*H_3_)_3_), N‐*C*‐N not observed. Elem. anal. calcd (%): N 7.20, C 49.37, H 7.51; found: N 7.77, C 48.98, H 7.51. FTIR: $\tilde{v}$ (cm^−1^)=2960 (s, CH_3_), 1600 (s, C=O).

*[Ag(NHC)(dmm)]*: The starting material [Ag(NHC)(hmds)] (0.3 g, 0.66 mmol) is dissolved in 5 mL of diethyl ether and dimethyl propanedioate (0.068 g, 0.66 mmol) is slowly added to the solution at RT which is stirred for 1 h. The solvent was removed under vacuum and exchanged by 10 mL THF after which the resulting clear solution was concentrated and stored at −35 °C overnight to afford colorless crystals. Yield: 0.165 g (35 %). ^1^H NMR (200 MHz, C_6_D_6_): *δ*=4.37 (s, O=C−C*H*−C=O), 3.66 (s, N−C*H_2_*−C*H_2_*−N), 2.89 (s, O=C−O−CH_3_), 1.21 ppm (s, NC(C*H*_3_)_3_). ^13^C NMR (50 MHz, C_6_D_6_): *δ*=172.6 (s, O=*C*−CH−*C*=O), 54.9 N*C*(CH_3_)_3_), 49.6 (s, O=C−O−*C*H_3_)), 45.8 (s, N−*C*H_2_−*C*H_2_−N), 40.8 (s, O=C−*C*H−C=O), 30.6 ppm (s, NC(*C*H_3_)_3_), N−*C*−N not observed. Elem. anal. calcd (%): N 6.65, C 45.62, H 6.94; found: N 6.96, C 45.08, H 6.98. FTIR: $\tilde{v}$ (cm^−1^)=2960 (s, CH_3_), 1666 to 1718 (d, C=O).

*[Ag(NHC)(fod)]*: The starting material [Ag(NHC)(hmds)] (0.5 g, 1.11 mmol) is dissolved in 10 mL of hexane and 2,2‐dimethyl‐6,6,7,7,8,8,8‐heptafluorooctane‐3,5‐dione (0.33 g, 1.11 mmol) is slowly added to the solution at RT which is stirred overnight. The resulting colorless suspension is mildly heated to completely dissolve the solid and stored at −35 °C overnight to afford colorless crystals which are dried under vacuum. Yield: 0.59 g (89 %). ^1^H NMR (200 MHz, C_6_D_6_): *δ* (ppm)=6.20 (t, *J*=1.8 Hz, O=C−C*H*−C=O), 2.82 (s, N−C*H_2_*−C*H_2_*−N), 1.25 (s, O=CC(C*H*_3_)_3_), 1.19 (s, NC(C*H*_3_)_3_), slight impurities observed at 1.02 and 2.61 ppm. ^13^C NMR (50 MHz, C_6_D_6_): *δ*=205.6 (s, N−*C*−N or O=*C*C(CH_3_)_3_)), 204.3 (s, N−*C*−N or O=*C*C(CH_3_)_3_), 172.8 to 172.0 (m, O=*C*−CF_2_−CF_2_−CF_3_), 89.9 (O=C−*C*H−C=O), 54.9 (s, N−*C*H_2_−*C*H_2_−N), 45.4 (s, N*C*(CH_3_)_3_), 42.7 (s, O=C*C*(CH_3_)_3_), 30.3 (s, NC(*C*H_3_)_3_), 28.3 (s, (CC(*C*H_3_)_3_), slight impurities observed at 27.5 ppm, −*C*F_2_−*C*F_2_−*C*F_3_ not observed. Elem. anal. calcd (%): N 4.79, C 43.09, H 5.51; found: N 4.98, C 42.73, H 5.53. FTIR: $\tilde{v}$ (cm^−1^)=2960 (s, CH_3_), 1630 (s, C=O).

*[Ag(NHC)(hfac)]*: The starting material [Ag(NHC)(hmds)] (0.6 g, 0.66 mmol) is dissolved in 5 mL of diethyl ether and 1,1,1,5,5,5‐hexafluoropentane‐2,4‐dione (0.14 g, 0.66 mmol) is slowly added to the solution at RT which is stirred overnight. The resulting colorless solution is concentrated and stored at −35 °C overnight to afford colorless crystals which are dried under vacuum. Yield: 0.21 g (61 %). ^1^H NMR (200 MHz, C_6_D_6_): *δ*=6.35 (m, O=C−C*H*−C=O), 2.68 (s, N−C*H_2_*−C*H_2_*−N), 1.10 (NC(C*H*_3_)_3_), slight impurities observed at 0.93 and 2.58 ppm. ^13^C NMR (50 MHz, C_6_D_6_): *δ*=202.5 (s, N−*C*−N), 177.3 (m, O=*C*−CH−*C*=O), 121.7 (s, *C*F_3_), 87.7 (m, O=C−*C*H−C=O), 54.8 (s, N−*C*H_2_−*C*H_2_−N), 45.4 (s, N*C*(CH_3_)_3_), 30.2 (s, NC(*C*H_3_)_3_), slight impurities observed at 115.93 and 27.5 ppm. Elem. anal. calcd (%): N 5.63, C 38.65, H 4.66; found: N 5.77, C 38.39, H 4.60. FTIR: $\tilde{v}$ (cm^−1^)=2960 (s, CH_3_), 1624 to 1656 (d, C=O).

## Conflict of interest

The authors declare no conflict of interests.

1

## Supporting information

As a service to our authors and readers, this journal provides supporting information supplied by the authors. Such materials are peer reviewed and may be re‐organized for online delivery, but are not copy‐edited or typeset. Technical support issues arising from supporting information (other than missing files) should be addressed to the authors.

Supporting Information

## Data Availability

The data that support the findings of this study are available in the supplementary material of this article.
