# Supplementary material for: Role of Anionic Backbone in NHC‐Stabilized Coinage Metal Complexes: New Precursors for Atomic Layer Deposition
Source: Chemistry. 2022 Feb 15;28(16):e202103798. doi: 10.1002/chem.202103798 (PMC9303662; doi:10.1002/chem.202103798)
Supplement: Supplementary file 1 — Supporting Information [file CHEM-28-0-s001.pdf]

Chemistry–A European Journal

Supporting Information

Role of Anionic Backbone in NHC-Stabilized Coinage Metal Complexes: New Precursors for Atomic Layer Deposition

Nils Boysen, Anish Philip, Detlef Rogalla, Maarit Karppinen, and Anjana Devi*

S1.Analysis of the Precursors

^{1}H-NMR Analysis

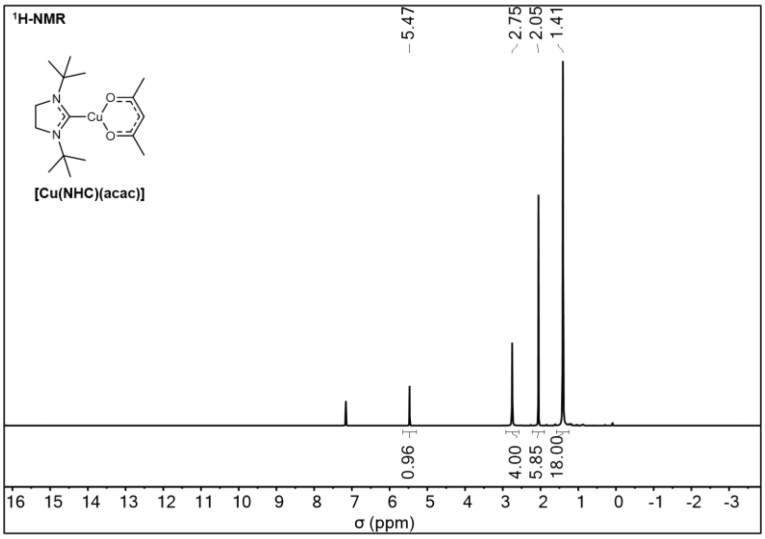

Figure S 1. ^{1}H-NMR spectrum of [Cu(NHC)(acac)] measured in C_6D_6.

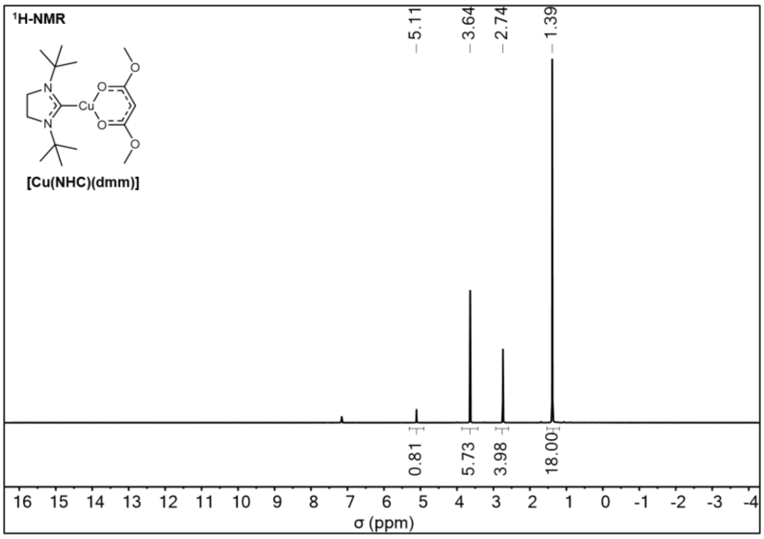

Figure S 2. ^{1}H-NMR spectrum of [Cu(NHC)(dmm)] measured in C_6D_6.

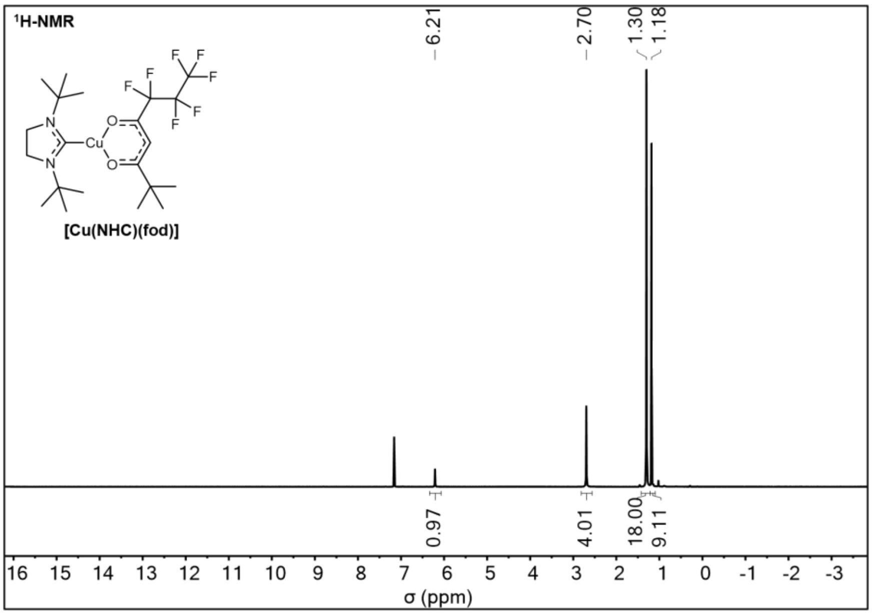

Figure S 3. ^{1}H-NMR spectrum of [Cu(NHC)(fod)] measured in C_6D_6.

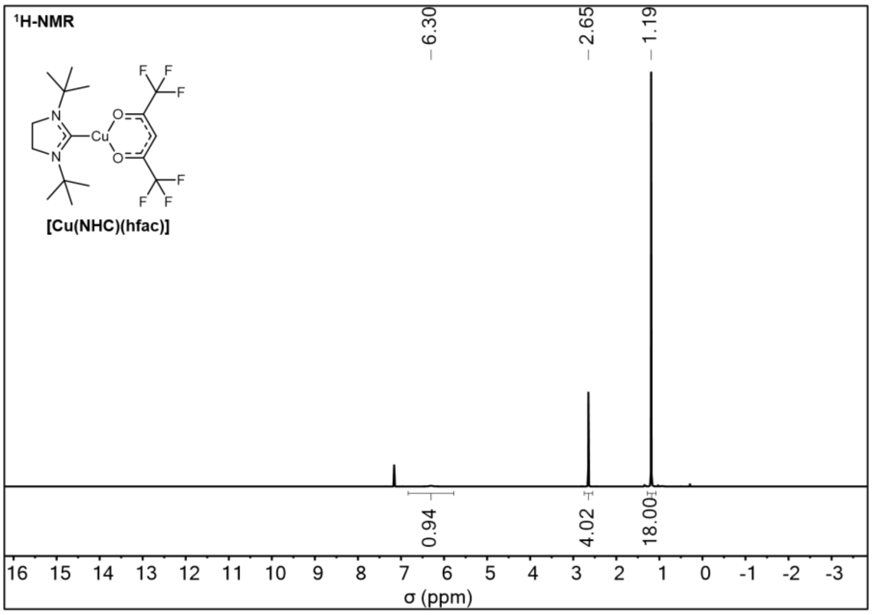

Figure S 4. ^{1}H-NMR spectrum of [Cu(NHC)(hfac)] measured in C_6D_6.

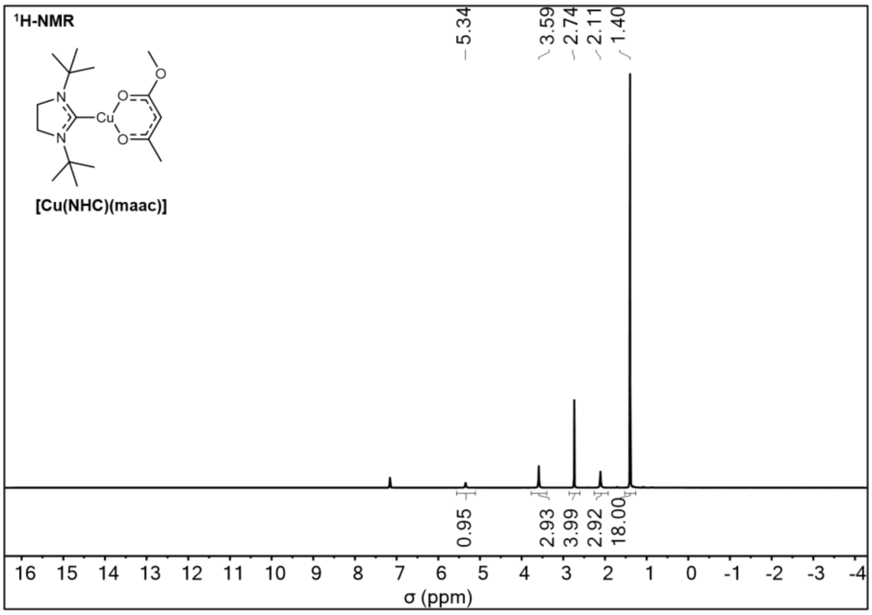

Figure S 5. ^{1}H-NMR spectrum of [Cu(NHC)(maac)] measured in C_6D_6.

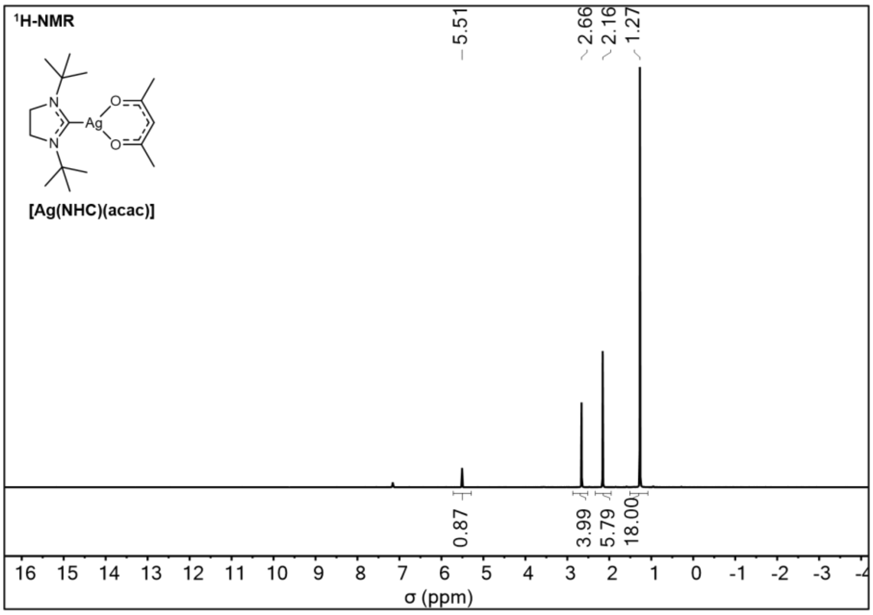

Figure S 6. ^{1}H-NMR spectrum of [Ag(NHC)(acac)] measured in C_6D_6.

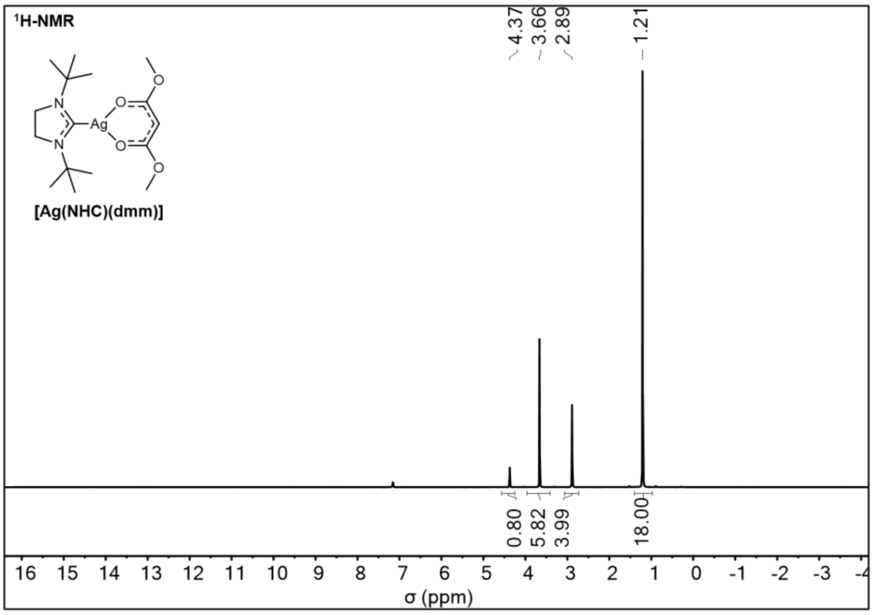

Figure S 7. ^{1}H-NMR spectrum of [Ag(NHC)(dmm)] measured in C_6D_6.

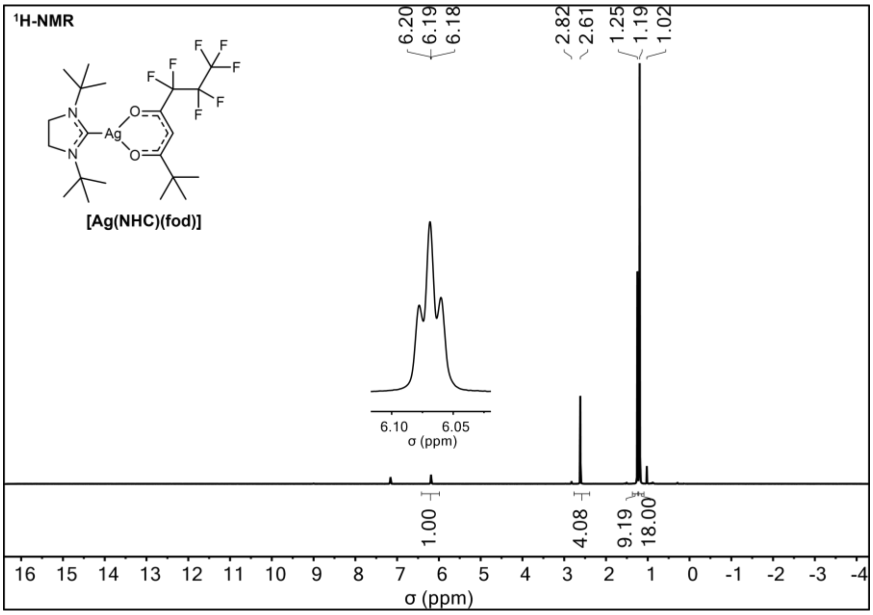

Figure S 8. ^{1}H-NMR spectrum of [Ag(NHC)(fod)] measured in C_6D_6.

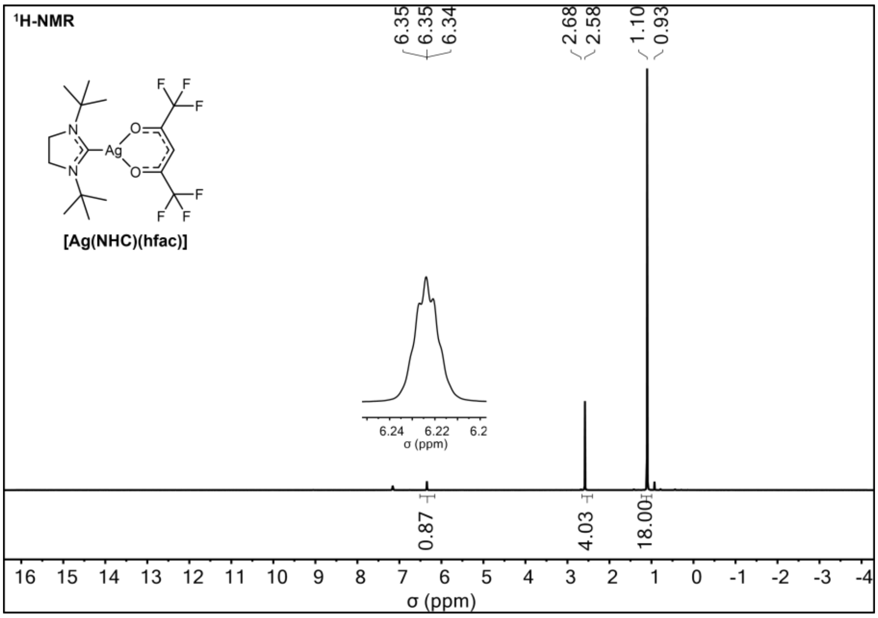

Figure S 9. ^{1}H-NMR spectrum of [Ag(NHC)(hfac)] measured in C_6D_6.

^{13}C-NMR Analysis

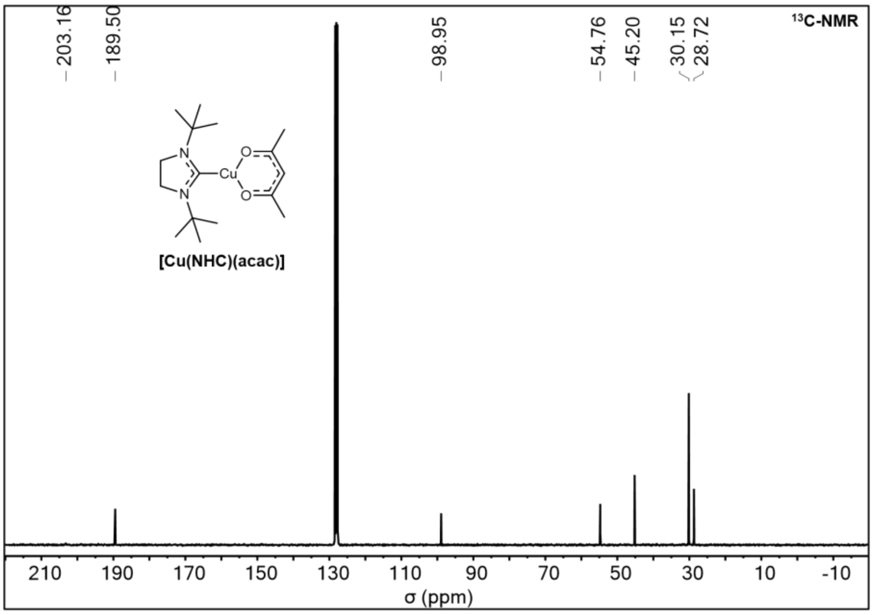

Figure S 10. ^{13}C-NMR spectrum of [Cu(NHC)(acac)] measured in C_6D_6.

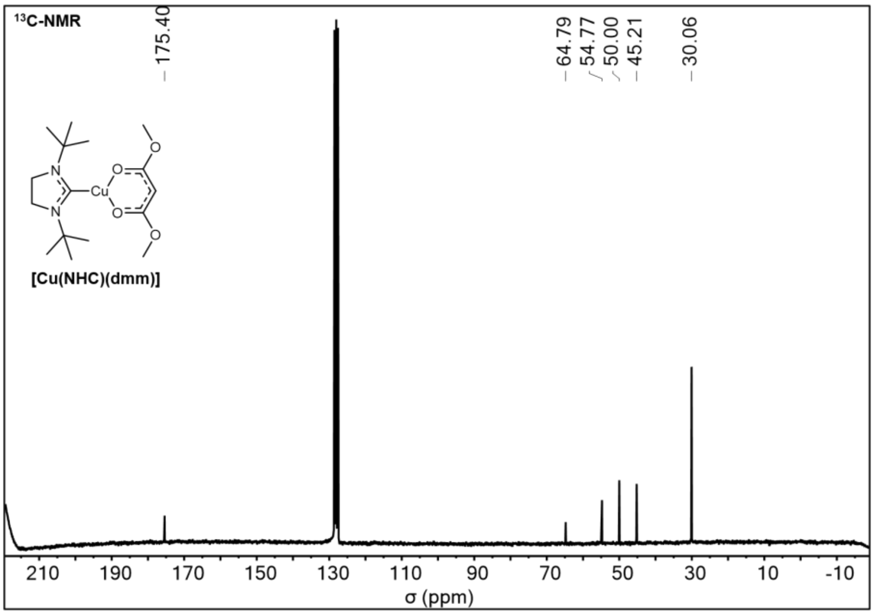

Figure S 11. [13]*C-NMR spectrum of [Cu(NHC)(dmm)] measured in* C_6D_6.

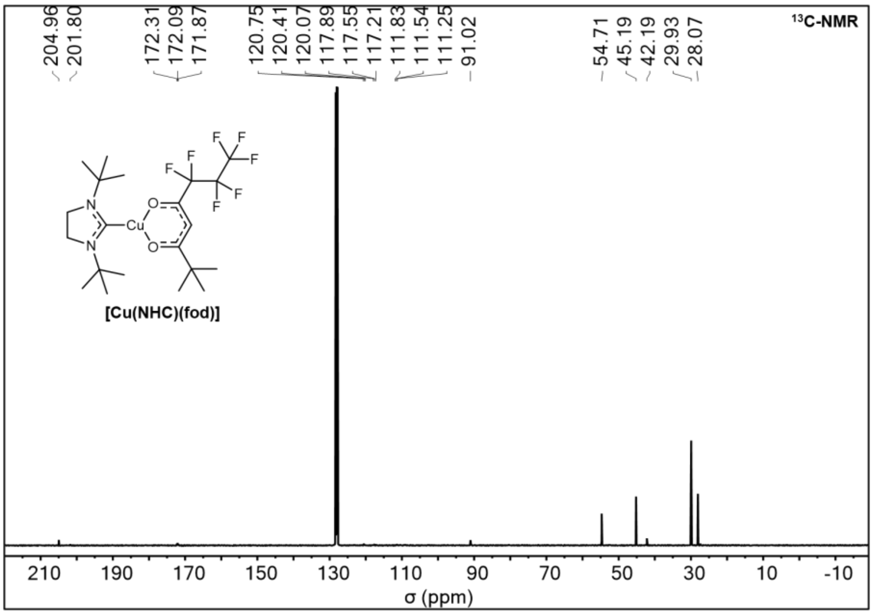

Figure S 12. [13]*C-NMR spectrum of [Cu(NHC)(fod)] measured in* C_6D_6.

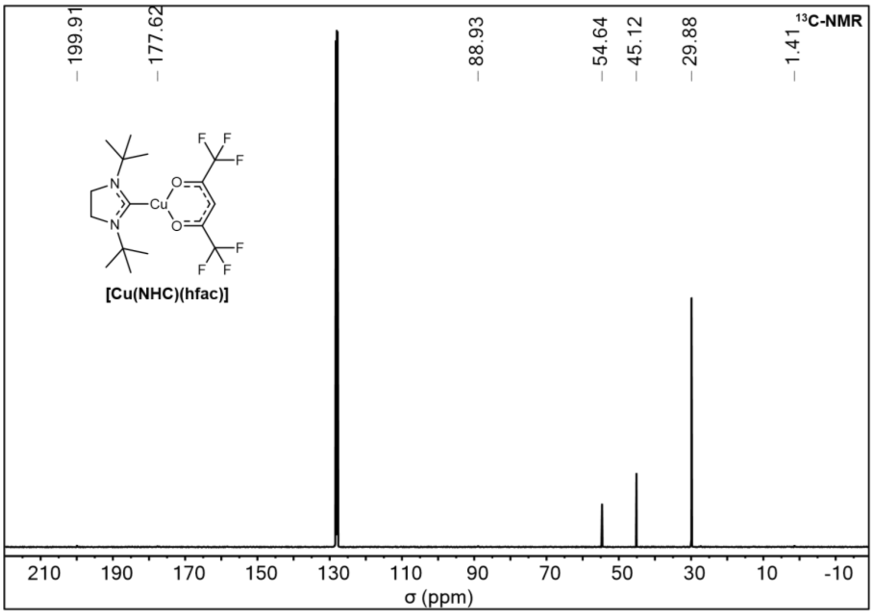

Figure S 13. ^{13}C-NMR spectrum of [Cu(NHC)(hfac)] measured in C_6D_6.

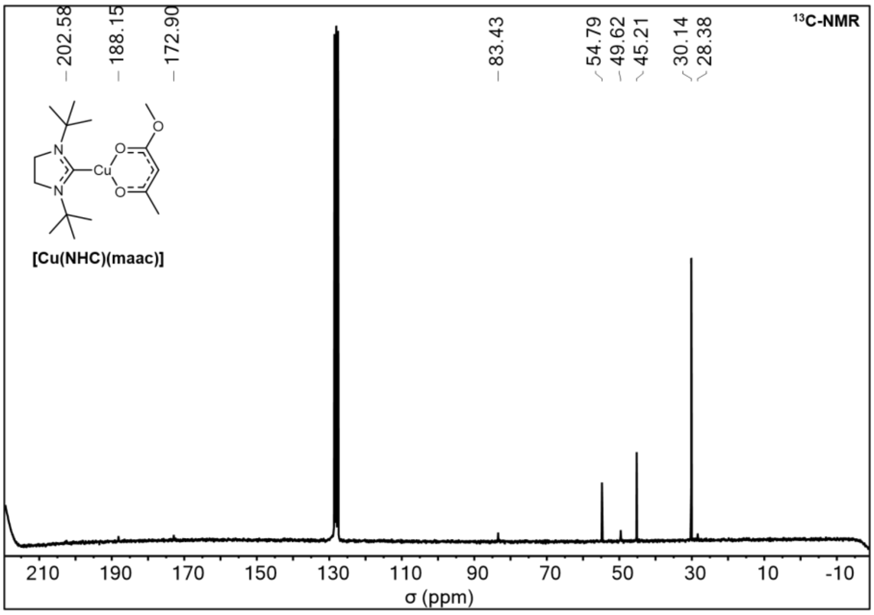

Figure S 14. ^{13}C-NMR spectrum of [Cu(NHC)(maac)] measured in C_6D_6.

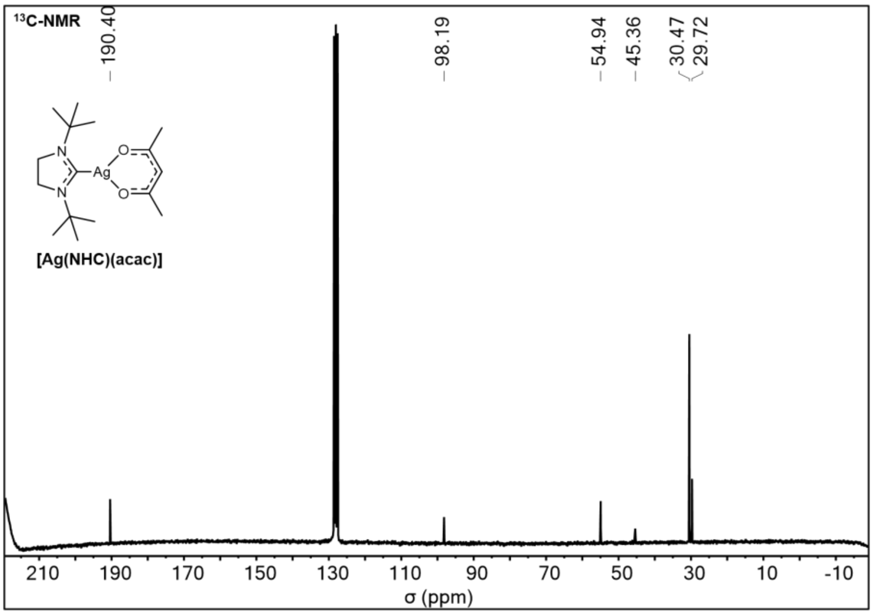

Figure S 15. ^{13}C-NMR spectrum of [Ag(NHC)(acac)] measured in C_6D_6.

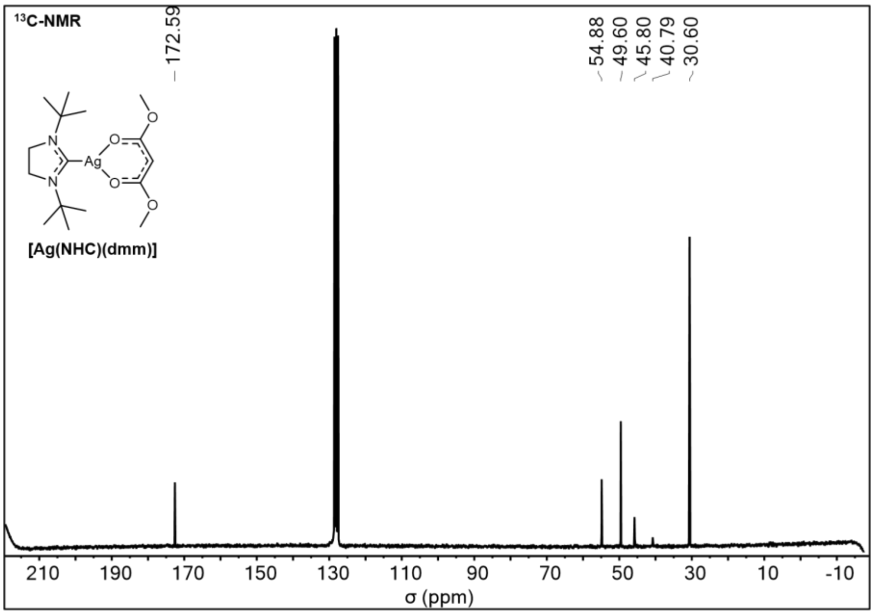

Figure S 16. ^{13}C-NMR spectrum of [Ag(NHC)(dmm)] measured in C_6D_6.

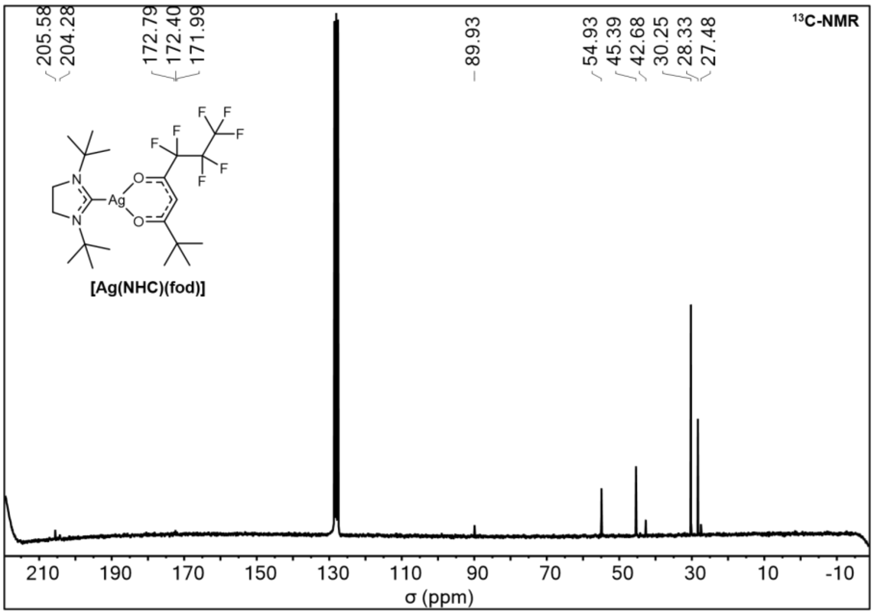

Figure S 17. ^{13}C-NMR spectrum of [Ag(NHC)(fod)] measured in C_6D_6.

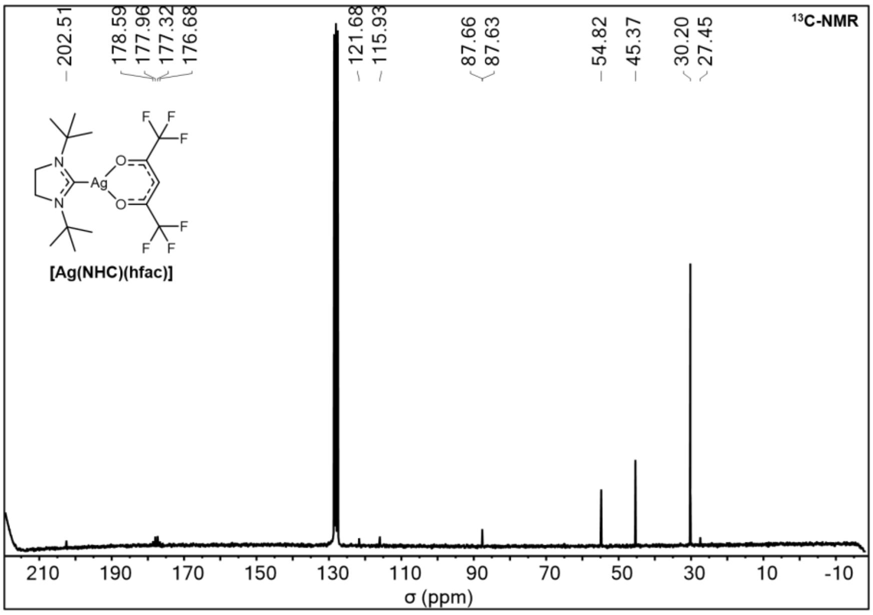

Figure S 18. ^{13}C-NMR spectrum of [Ag(NHC)(hfac)] measured in C_6D_6.

EI-MS

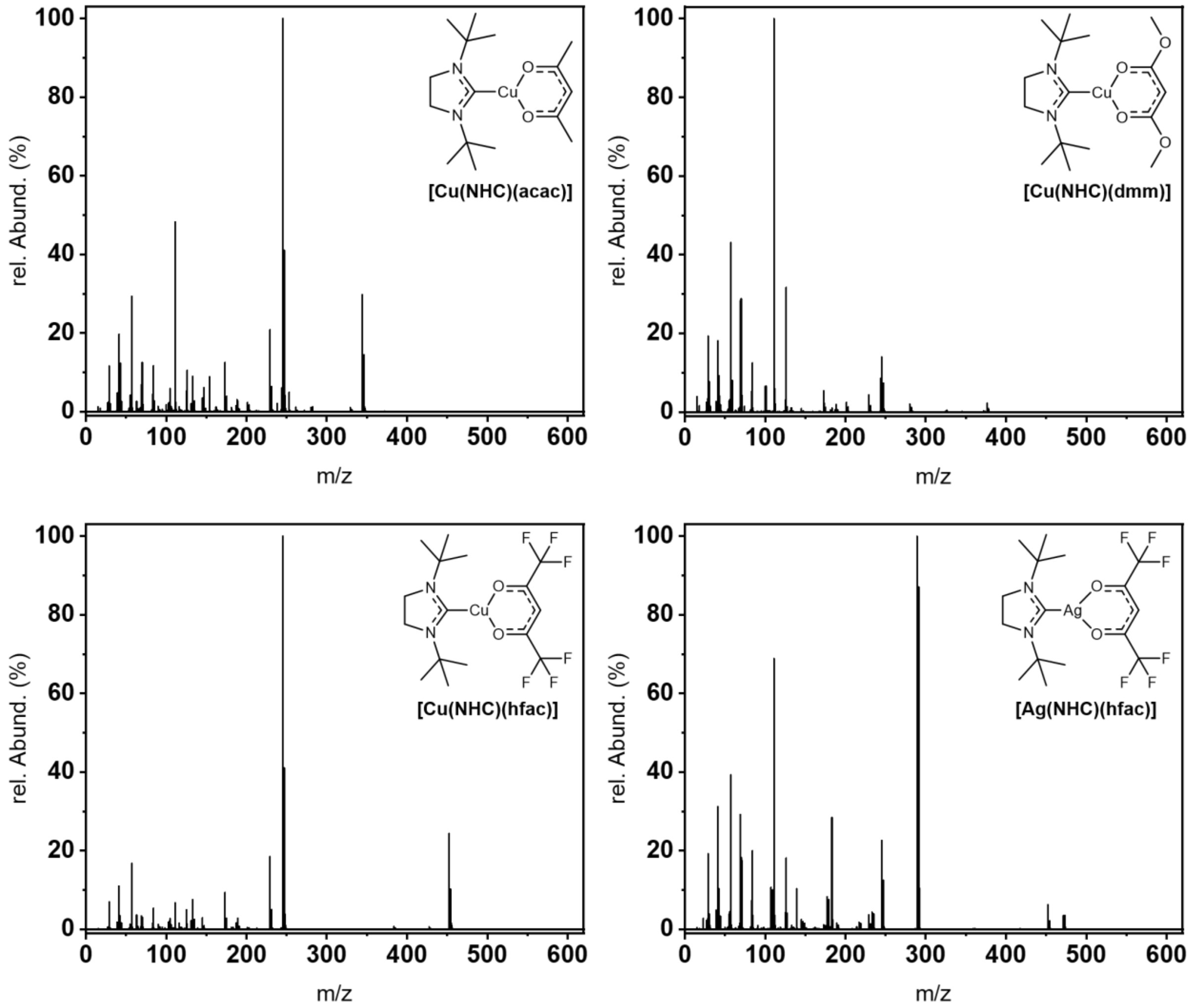

Figure S 19. EI-MS graphs for the complexes [Cu(NHC)(acac)], [Cu(NHC)(dmm)], [Cu(NHC)(hfac)] and [Ag(NHC)(hfac)].

FT-IR

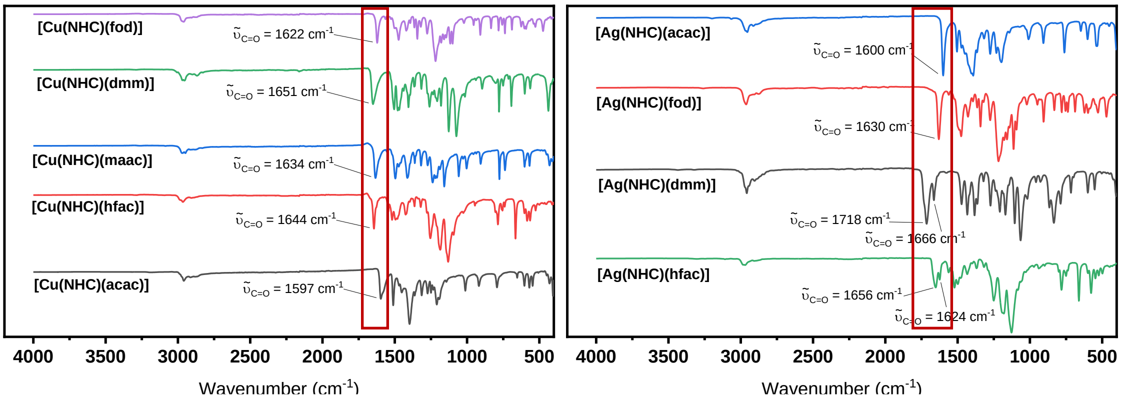

Figure S 20. FT-IR spectra of all Cu and Ag compounds synthesized in this study with the symmetric stretching band of the C=O bond highlighted in a red box.

<u>SC-XRD</u>

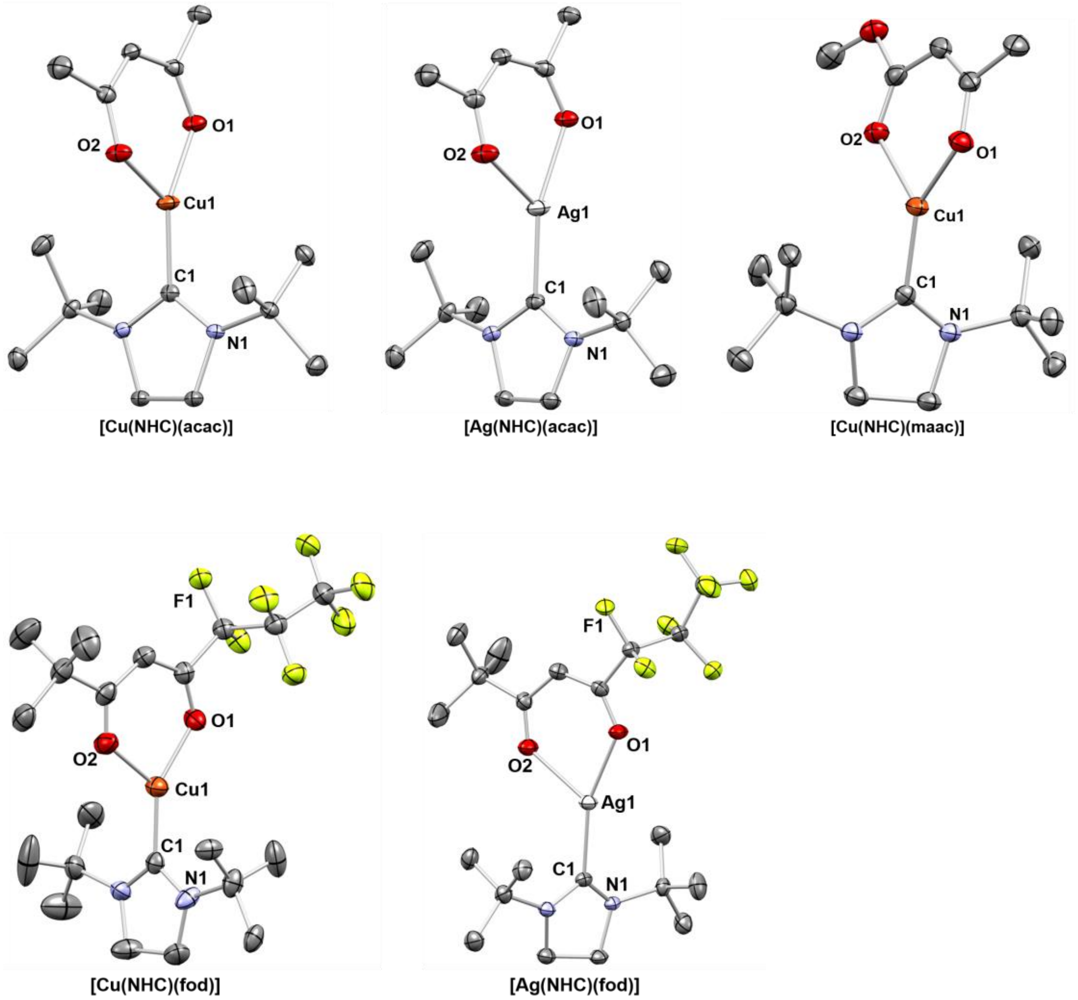

Figure S 21. Illustrations of SC-XRD structures for the remaining Cu and Ag complexes. Disorders are omitted for clarity, while the thermal ellipsoids are drawn at a probability level of 50 %.

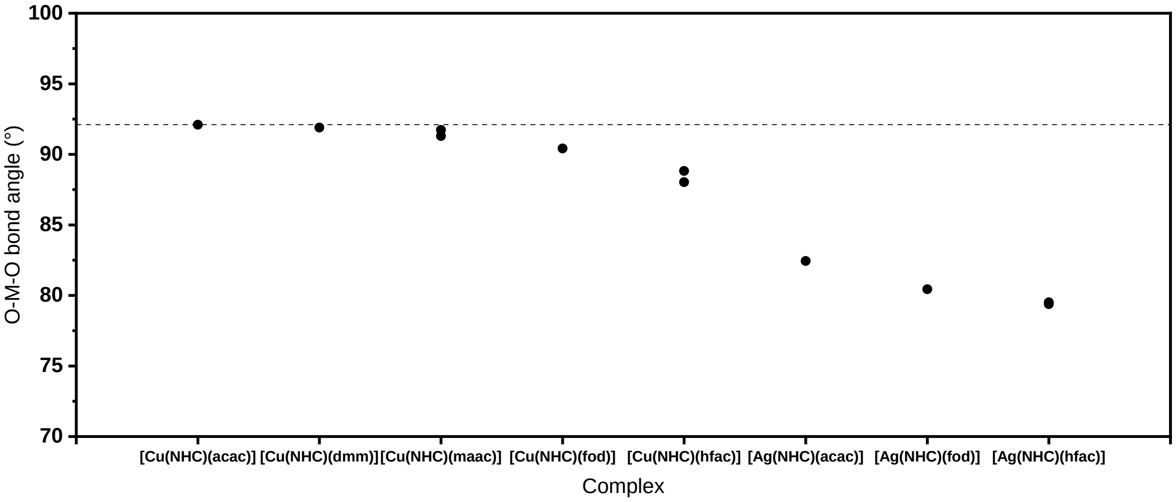

Figure S 22. O-M-O bond angles obtained from SC-XRD measurements for all evaluated complexes.

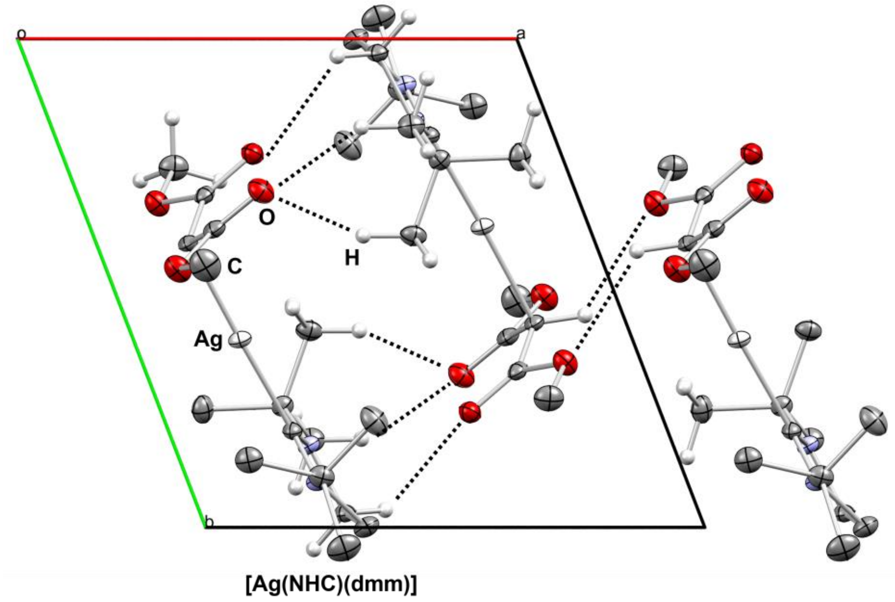

Figure S 23. Molecular packing of [Ag(NHC)(dmm)] in the solid crystalline state with a view along the c-axis.

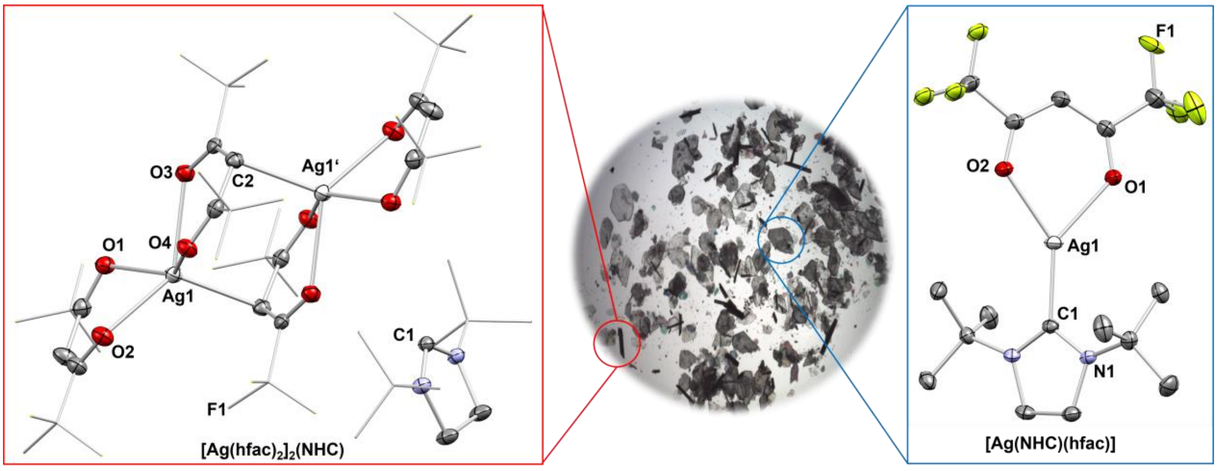

Figure S 24. Microscopic view of the crystalline mixture obtained after isolating the product [Ag(NHC)(hfac)]. A second crystalline species was identified as needle-shaped crystals. The molecular structure was evaluated to be a dimeric ion with [Ag(hfac)$_2$]$_2$(NHC)$_2$

Table S1 Crystal data and structure refinement for the copper precursor complexes.

Identification code	**[Cu(NHC)(acac)]**	**[Cu(NHC)(dmm)]**	**[Cu(NHC)(maac)]**	**[Cu(NHC)(fod)]**	**[Cu(NHC)(hfac)]**
Empirical formula	$C_{16}H_{29}N_2O_2Cu$	$C_{16}H_{29}N_2O_4Cu$	$C_{16}H_{29}N_2O_3Cu$	$C_{21}H_{32}F_7N_2O_2Cu$	$C_{16}H_{23}F_6N_2O_2Cu$
Formula weight	344.95	376.95	360.95	541.02	452.90
Temperature/K	100.00(10)	100.00(10)	102(3)	102(4)	100.00(10)
Crystal system	monoclinic	monoclinic	monoclinic	monoclinic	triclinic
Space group	$P2_1/n$	$P2_1/c$	$P2_1/c$	$P2_1/c$	P-1
a/Å	13.6668(2)	10.9728(2)	17.2809(2)	11.8629(2)	10.1106(4)
b/Å	9.56120(10)	15.3217(2)	11.29560(10)	10.2231(2)	11.2109(4)
c/Å	14.8975(2)	11.8646(2)	19.0884(2)	20.7184(3)	17.9060(7)
α/°	90	90	90	90	80.947(3)
β/°	116.993(2)	108.602(2)	101.3870(10)	95.9350(10)	88.467(3)
γ/°	90	90	90	90	73.553(3)
Volume/Å^3	1734.60(5)	1890.49(6)	3652.68(7)	2499.17(7)	1921.99(13)
Z	4	4	8	4	4
$\rho_{calc}g/cm^3$	1.321	1.324	1.313	1.438	1.565
μ/mm^{-1}	1.817	1.796	1.793	1.892	2.261
F(000)	736.0	800.0	1536.0	1120.0	928.0
Crystal size/mm^3	0.162 × 0.13 × 0.076	0.174 × 0.131 × 0.115	0.129 × 0.098 × 0.055	0.337 × 0.289 × 0.211	0.305 × 0.231 × 0.204
Radiation	Cu Kα (λ = 1.54184)	Cu Kα (λ = 1.54184)	Cu Kα (λ = 1.54184)	Cu Kα (λ = 1.54184)	Cu Kα (λ = 1.54184)
2Θ range for data collection/°	7.292 to 153.888	8.502 to 154.082	5.216 to 154.168	7.492 to 153.936	8.326 to 145.866
Index ranges	-14 ≤ h ≤ 17, -10 ≤ k ≤ 11, -18 ≤ l ≤ 18	-13 ≤ h ≤ 13, -16 ≤ k ≤ 19, -14 ≤ l ≤ 14	-21 ≤ h ≤ 21, -13 ≤ k ≤ 13, -17 ≤ l ≤ 24	-14 ≤ h ≤ 15, -12 ≤ k ≤ 11, -23 ≤ l ≤ 26	-12 ≤ h ≤ 10, -13 ≤ k ≤ 13, -22 ≤ l ≤ 21
Reflections collected	17073	13120	29197	17164	13151
Independent reflections	3573 [R_{int} = 0.0272, R_{sigma} = 0.0190]	3856 [R_{int} = 0.0318, R_{sigma} = 0.0308]	7406 [R_{int} = 0.0374, R_{sigma} = 0.0344]	5074 [R_{int} = 0.0325, R_{sigma} = 0.0335]	7441 [R_{int} = 0.0234, R_{sigma} = 0.0310]
Data/restraints/parameters	3573/0/198	3856/0/216	7406/0/413	5074/237/386	7441/0/499
Goodness-of-fit on F^2	1.099	1.103	1.061	1.067	1.019
Final R indexes [I>=2σ (I)]	R_1 = 0.0281, wR_2 = 0.0761	R_1 = 0.0340, wR_2 = 0.0920	R_1 = 0.0382, wR_2 = 0.1013	R_1 = 0.0396, wR_2 = 0.1009	R_1 = 0.0319, wR_2 = 0.0806
Final R indexes [all data]	R_1 = 0.0297, wR_2 = 0.0769	R_1 = 0.0373, wR_2 = 0.0939	R_1 = 0.0440, wR_2 = 0.1049	R_1 = 0.0456, wR_2 = 0.1045	R_1 = 0.0389, wR_2 = 0.0854
Largest diff. peak/hole / e Å^{-3}	0.39/-0.48	0.49/-0.48	0.87/-0.75	0.40/-0.31	0.39/-0.35

Table S2 Crystal data and structure refinement for the silver precursor complexes.

Identification code	**[Ag(NHC)(acac)]**	**[Ag(NHC)(dmm)]**	**[Ag(NHC)(fod)]**	**[Ag(NHC)(hfac)]**
Empirical formula	$C_{16}H_{29}N_2O_2Ag$	$C_{16}H_{29}AgN_2O_4$	$C_{21}H_{32}F_7N_2O_2Ag$	$C_{16}H_{23}F_6N_2O_2Ag$
Formula weight	389.28	421.28	585.35	497.23
Temperature/K	100.00(10)	100.01(10)	100.00(10)	100.00(10)
Crystal system	monoclinic	triclinic	monoclinic	monoclinic
Space group	$P2_1/n$	P-1	$P2_1/c$	$P2_1/c$
a/Å	13.6487(2)	9.6714(3)	11.9849(3)	19.2507(6)
b/Å	9.73280(10)	10.2054(4)	9.9847(2)	11.5297(3)
c/Å	14.7556(2)	10.4454(2)	20.8254(4)	19.7079(6)
α/°	90	79.122(2)	90	90
β/°	115.544(2)	81.548(2)	97.135(2)	116.977(4)
γ/°	90	67.842(3)	90	90
Volume/$Å^3$	1768.54(5)	934.41(5)	2472.79(9)	3898.3(2)
Z	4	2	4	8
$\rho_{calc}g/cm^3$	1.462	1.497	1.572	1.694
μ/mm^{-1}	9.185	8.823	7.206	8.947
F(000)	808.0	436.0	1192.0	2000.0
Crystal size/mm^3	0.189 × 0.149 × 0.112	0.115 × 0.099 × 0.08	0.124 × 0.115 × 0.081	0.181 × 0.132 × 0.097
Radiation	Cu Kα (λ = 1.54184)	Cu Kα (λ = 1.54184)	Cu Kα (λ = 1.54184)	Cu Kα (λ = 1.54184)
2Θ range for data collection/°	7.384 to 155.044	8.65 to 154.934	7.434 to 148.09	8.994 to 146.102
Index ranges	-17 ≤ h ≤ 17, -11 ≤ k ≤ 12, -18 ≤ l ≤ 18	-11 ≤ h ≤ 12, -11 ≤ k ≤ 12, -13 ≤ l ≤ 13	-14 ≤ h ≤ 13, -12 ≤ k ≤ 8, -24 ≤ l ≤ 25	-23 ≤ h ≤ 23, -13 ≤ k ≤ 13, -13 ≤ l ≤ 23
Reflections collected	22287	23046	9716	14825
Independent reflections	3700 [R_{int} = 0.0419, R_{sigma} = 0.0245]	3902 [R_{int} = 0.0483, R_{sigma} = 0.0284]	4844 [R_{int} = 0.0343, R_{sigma} = 0.0477]	7536 [R_{int} = 0.0359, R_{sigma} = 0.0452]
Data/restraints/parameters	3700/0/198	3902/0/216	4844/111/338	7536/266/557
Goodness-of-fit on F^2	1.098	1.135	1.055	1.029
Final R indexes [I>=2σ (I)]	R_1 = 0.0268, wR_2 = 0.0711	R_1 = 0.0230, wR_2 = 0.0589	R_1 = 0.0322, wR_2 = 0.0773	R_1 = 0.0364, wR_2 = 0.0911
Final R indexes [all data]	R_1 = 0.0278, wR_2 = 0.0718	R_1 = 0.0236, wR_2 = 0.0592	R_1 = 0.0425, wR_2 = 0.0824	R_1 = 0.0468, wR_2 = 0.0985
Largest diff. peak/hole / e $Å^{-3}$	0.68/-0.94	0.47/-0.57	0.59/-0.58	1.12/-0.82

Table** S3 **Crystal data and structure refinement for the copper and silver precursor complexes.

	**C1–M (Å)**	**O1–M (Å)**	**O2–M (Å)**	**O1–C (Å)**	**O2-C (Å)**	**O1–M–O2 (°)**
[Cu(NHC)(acac)]	1.8877(14)	1.9693(11)	2.0509(11)	1.2656(17)	1.2649(18)	92.10(4)
[Cu(NHC)(dmm)]	1.8712(18)	1.9895(12)	2.0115(12)	1.251(2)	1.246(2)	91.90(5)
[Cu(NHC)(maac)]	1.8883(18),<br>1.8790(18)	1.9407(14),<br>1.9579(14)	2.1165(14),<br>2.0673(14)	1.242(2),<br>1.248(2)	1.275(2),<br>1.278(2)	91.30(6),<br>91.73(6)
[Cu(NHC)(fod)]	1.8889(19)	1.9665(14)	2.0482(15)	1.269(2)	1.245(3)	90.42(6)
[Cu(NHC)(hfac)]	1.8972(18),<br>1.8915(18)	2.0260(13),<br>2.0658(13)	2.0144(14),<br>2.0569(14)	1.250(2),<br>1.249(2)	1.250(2),<br>1.253(2)	88.03(5),<br>88.82(5)
[Ag(NHC)(acac)]	2.091(2)	2.1797(15)	2.3406(17)	1.251(3)	1.266(3)	82.44(6)
[Ag(NHC)(dmm)]	2.1087(19)	2.1752(19)*	-	1.210(3)	1.213(3)	-
[Ag(NHC)(fod)]	2.111(3)	2.183(2)	2.369(2)	1.265(4)	1.243(4)	80.44(8)
[Ag(NHC)(hfac)]	2.108(3),<br>2.101(3)	2.278(3),<br>2.335(2)	2.312(3),<br>2.335(2)	1.245(5),<br>1.248(4)	1.245(4),<br>1.239(5)	79.51(9),<br>79.38(9)

S2.Reactivity Studies

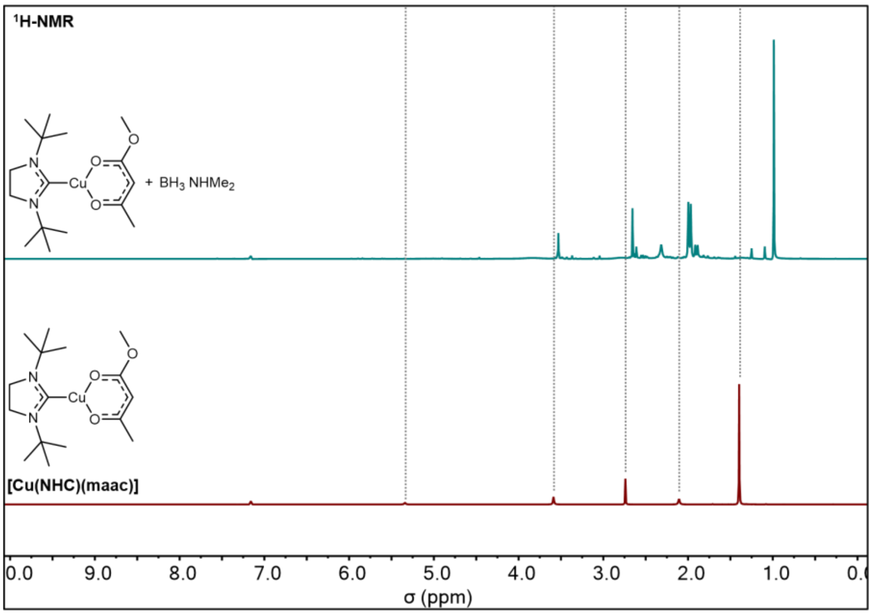

Figure S 25. Bottom: Initial ^{1}H-NMR spectrum of [Cu(NHC)(maac)]. Top: ^{1}H-NMR spectrum after a reaction with BH_3 $NHMe_2$.

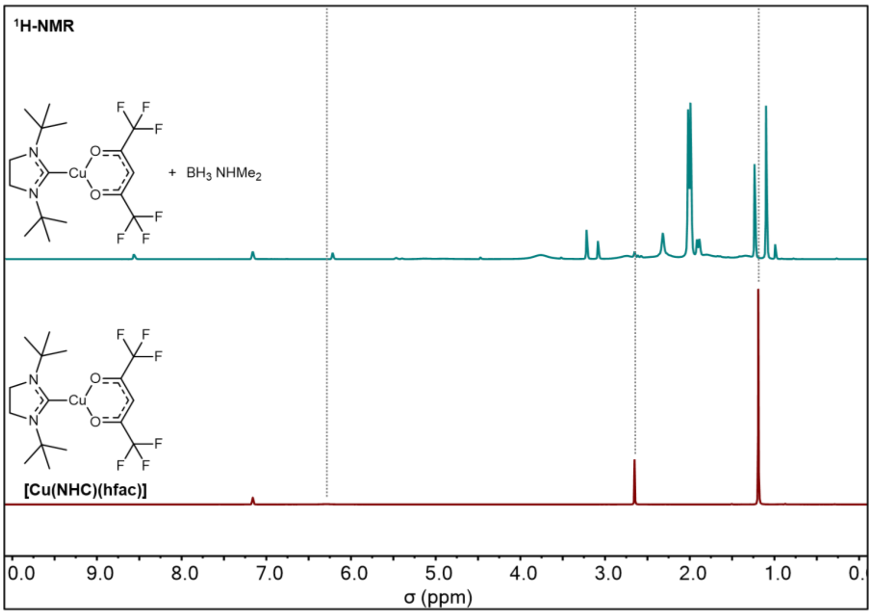

Figure S 26. Bottom: Initial ^{1}H-NMR spectrum of [Cu(NHC)(hfac)]. Top: ^{1}H-NMR spectrum after a reaction with BH_3 $NHMe_2$.

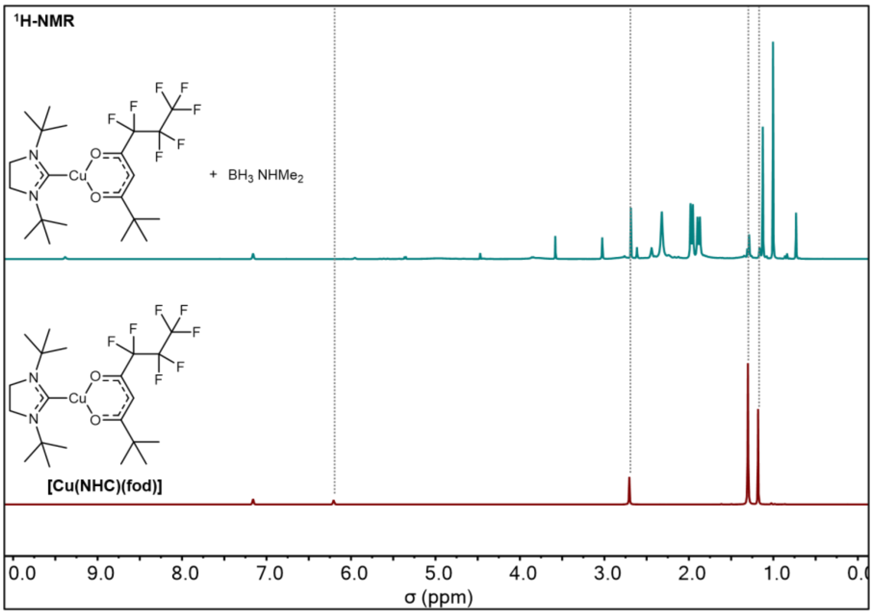

Figure S 27. Bottom: Initial ¹H-NMR spectrum of [Cu(NHC)(fod)]. Top: ¹H-NMR spectrum after a reaction with BH_3 $NHMe_2$.

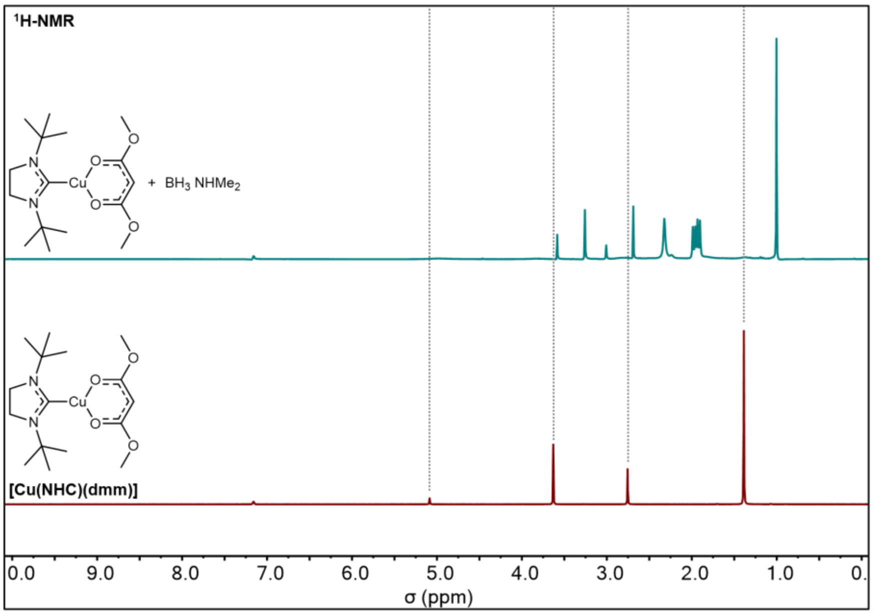

Figure S 28. Bottom: Initial ¹H-NMR spectrum of [Cu(NHC)(dmm)]. Top: ¹H-NMR spectrum after a reaction with BH_3 $NHMe_2$.

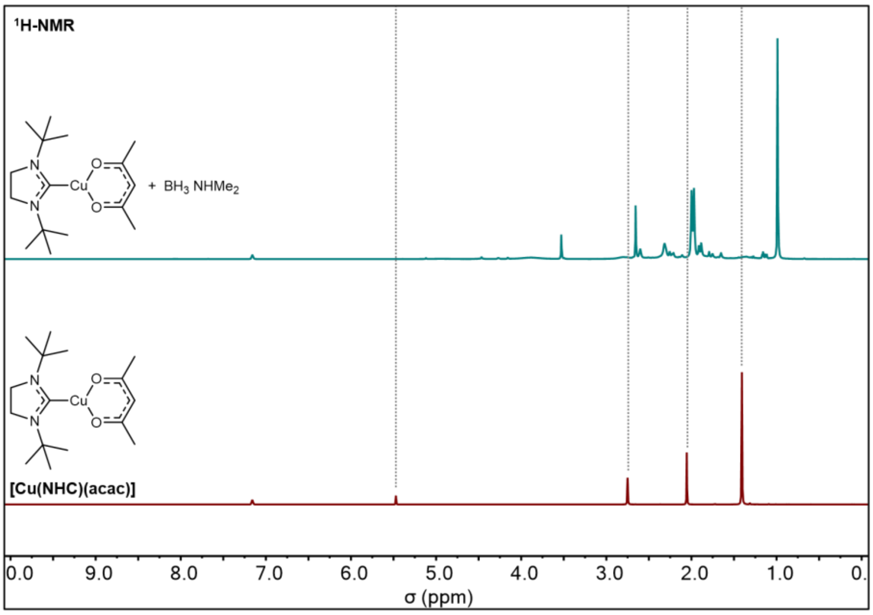

Figure S 29. Bottom: Initial [1]*H-NMR spectrum of [Cu(NHC)(acac)]. Top:* [1]*H-NMR spectrum after a reaction with* BH_3 $NHMe_2$*.*

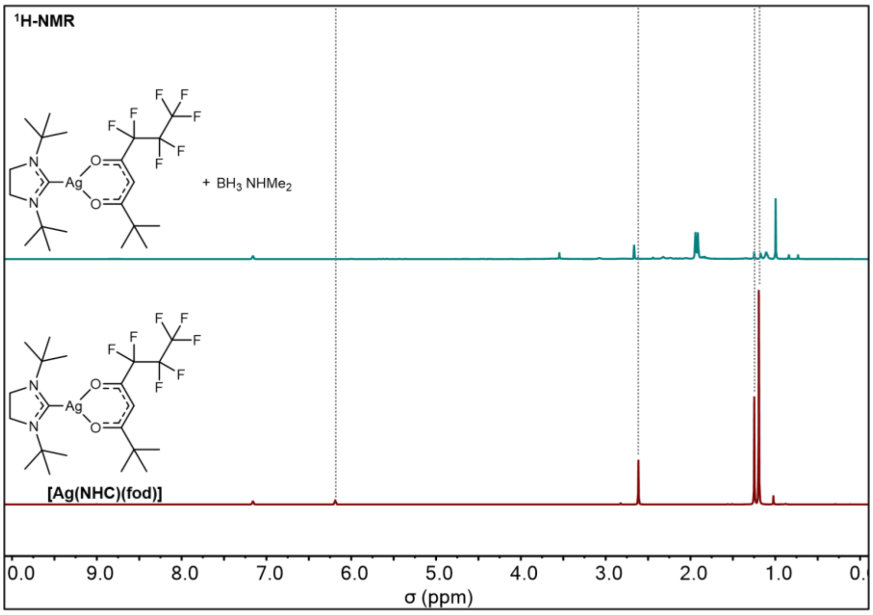

Figure S 30. Bottom: Initial [1]*H-NMR spectrum of [Ag(NHC)(fod)]. Top:* [1]*H-NMR spectrum after a reaction with* BH_3 $NHMe_2$*.*

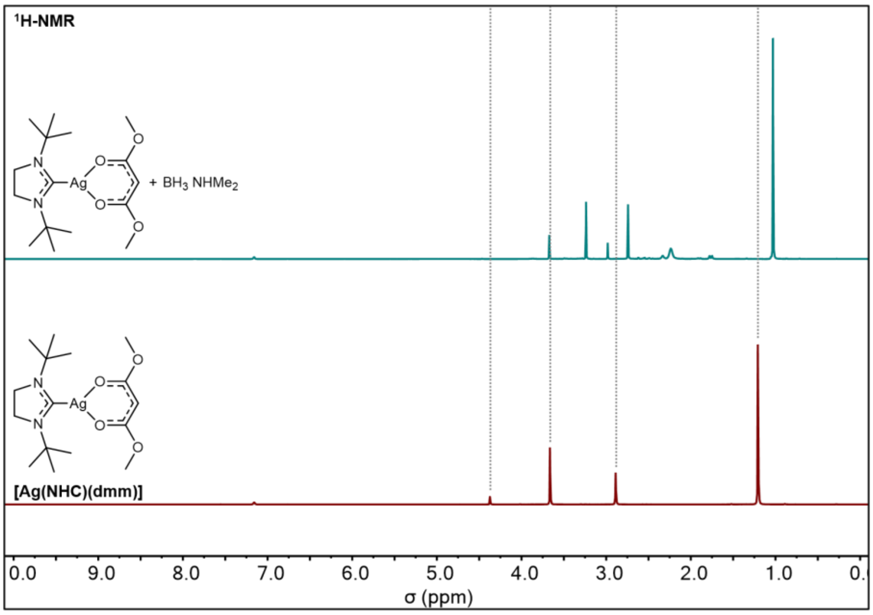

Figure S 31. Bottom: Initial [1]*H-NMR spectrum of [Ag(NHC)(dmm)]. Top:* [1]*H-NMR spectrum after a reaction with* BH_3 $NHMe_2$*.*

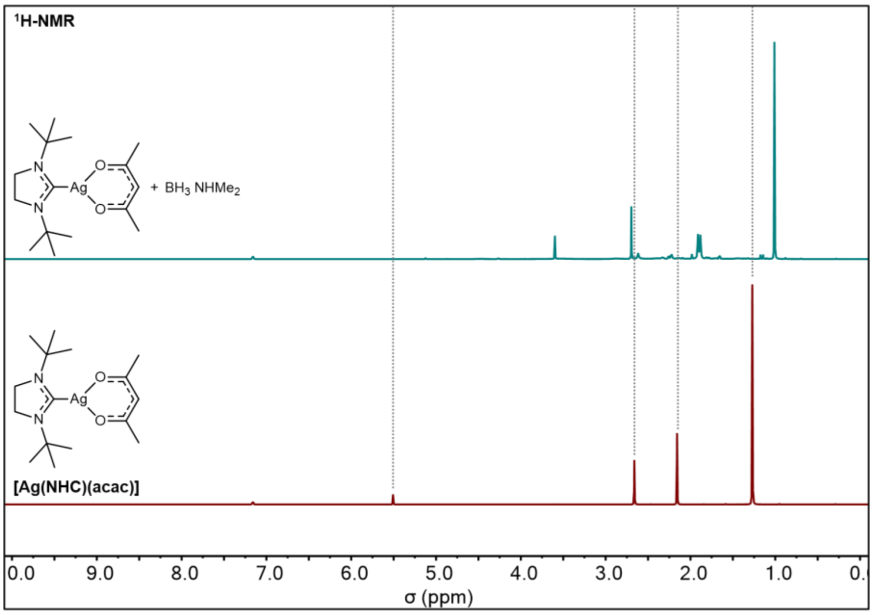

Figure S 32. Bottom: Initial [1]*H-NMR spectrum of [Ag(NHC)(acac)]. Top:* [1]*H-NMR spectrum after a reaction with* BH_3 $NHMe_2$*.*

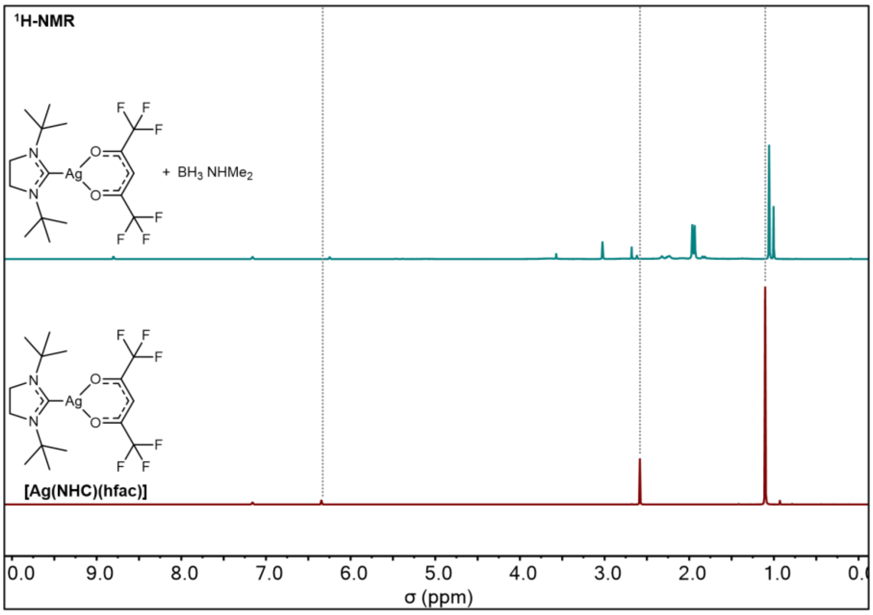

Figure S 33. Bottom: Initial ^{1}H-NMR spectrum of [Ag(NHC)(hfac)]. Top: ^{1}H-NMR spectrum after a reaction with BH_3 $NHMe_2$.

Vapor Pressure Measurements

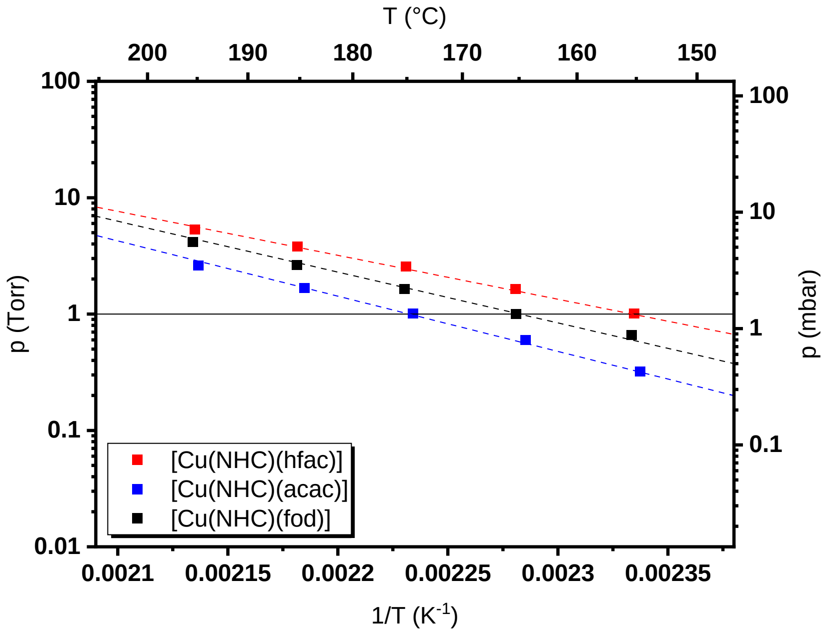

Figure S 34. Vapor pressure curves of [Cu(NHC)(hfac)], [Cu(NHC)(acac)] and [Cu(NHC)(fod)] obtained from stepped isothermal TGA measurements.

TG-DSC Analysis

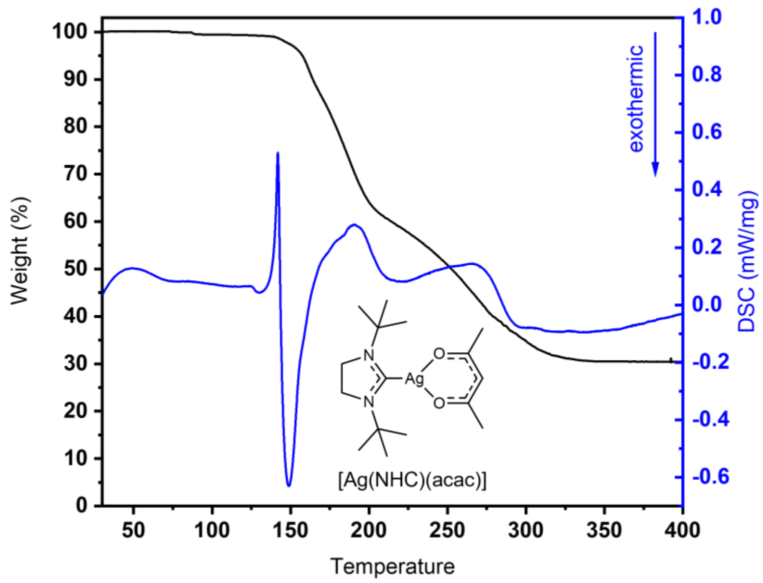

Figure S 35. TG (black) and DSC (blue) curves of [Ag(NHC)(acac)].

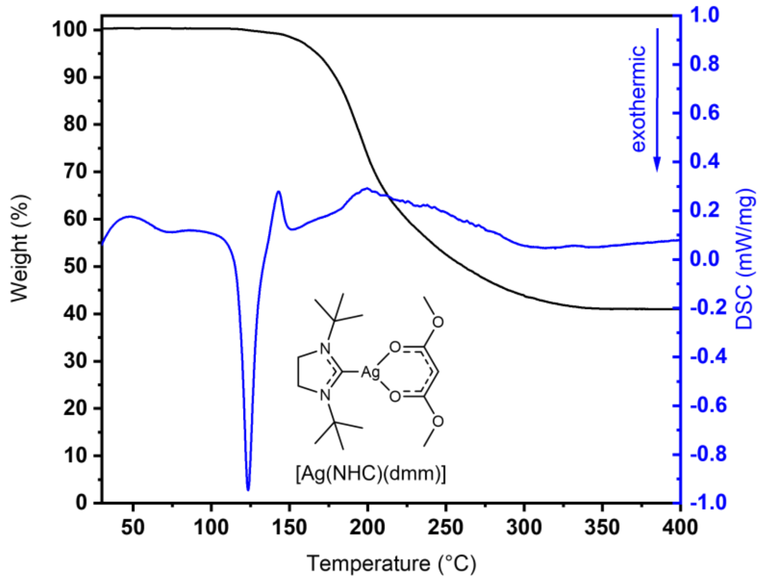

Figure S 36. TG (black) and DSC (blue) curves of [Ag(NHC)(dmm)].

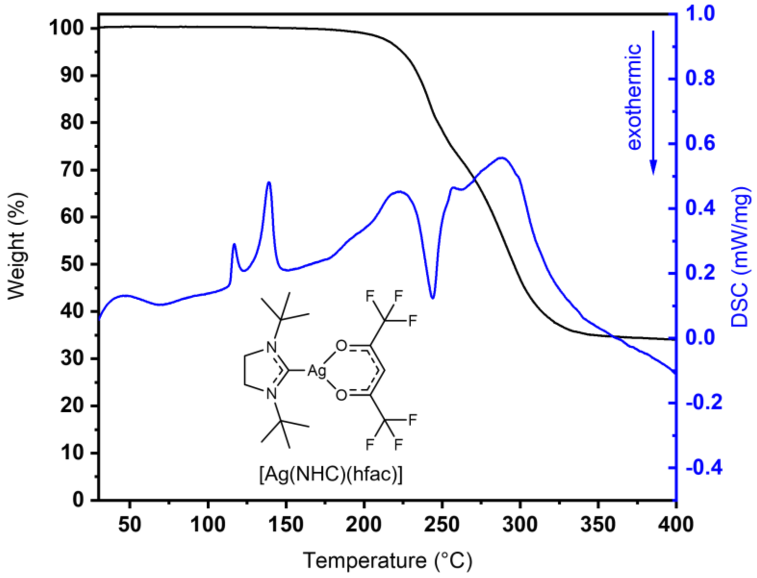

Figure S 37. TG (black) and DSC (blue) curves of [Ag(NHC)(hfac)].

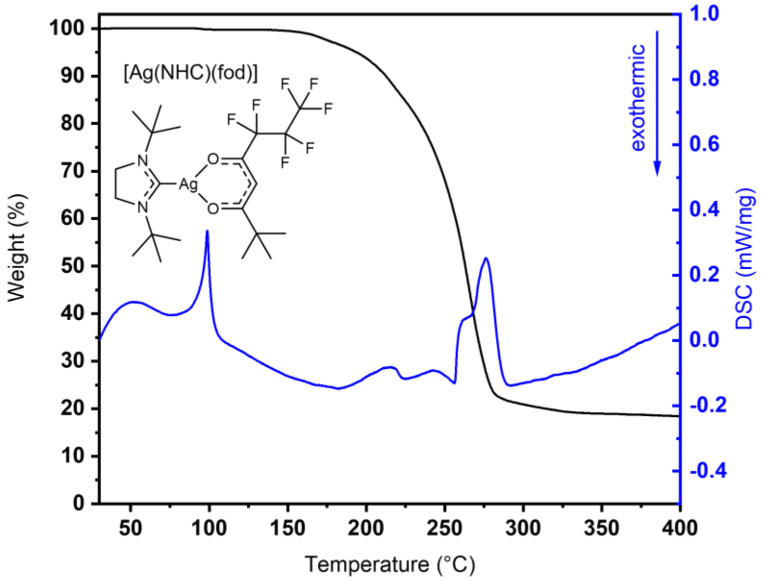

Figure S 38. TG (black) and DSC (blue) curves of [Ag(NHC)(fod)].

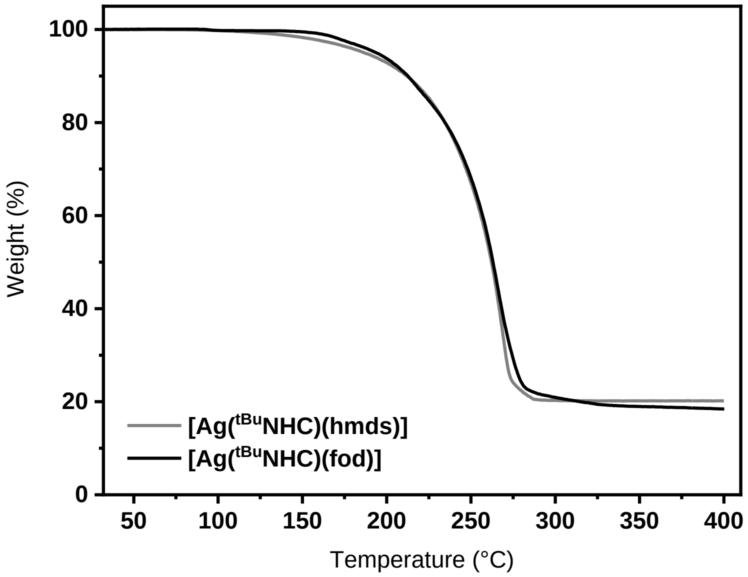

Figure S 39. TG curves of complexes [Ag(NHC)(hmds)] and [Ag(NHC)(fod)].[5]

RBS/NRA

Table S4. Results of the RBS/NRA measurements of the Cu deposits on Si substrates at a given deposition temperature (T_d).

T_d (°C)	Cu (10^{15} atoms/cm^2)	Cu (at.%)	C (at.%)	N (at.%)	O (at.%)
**145 °C**	66.3	76.7	15.0	1.9	6.4
**160 °C**	42.0	80.9	8.8	3.5	6.9

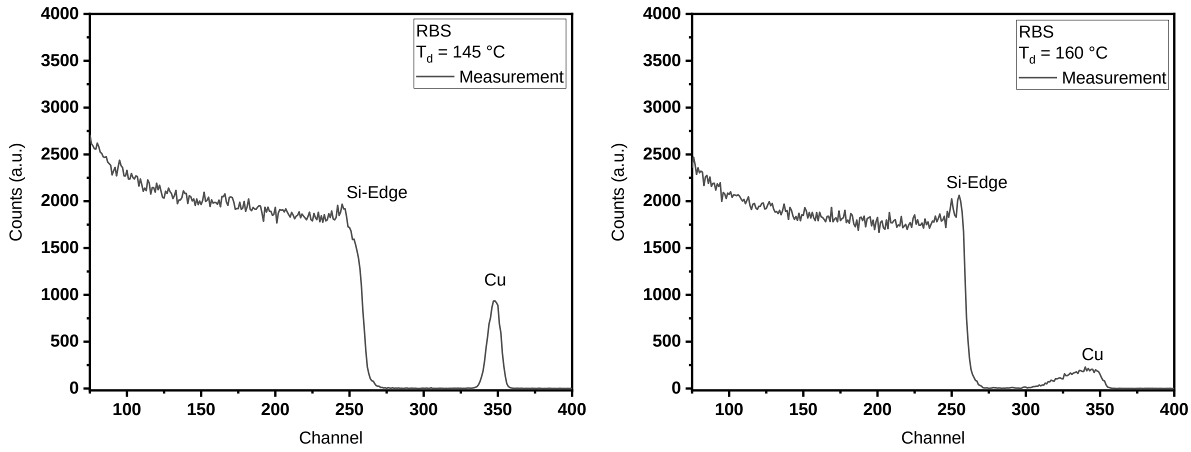

Figure S 40. RBS spectrua of the of the Cu deposits on Si substrates at a given deposition temperature (T_d).For T_d = 145 °C the calibration offset is -41.1 keV and energy per channel is 4.52 keV. For T_d = 160 °C the calibration offset is -45.0 keV and energy per channel is 4.53 keV.

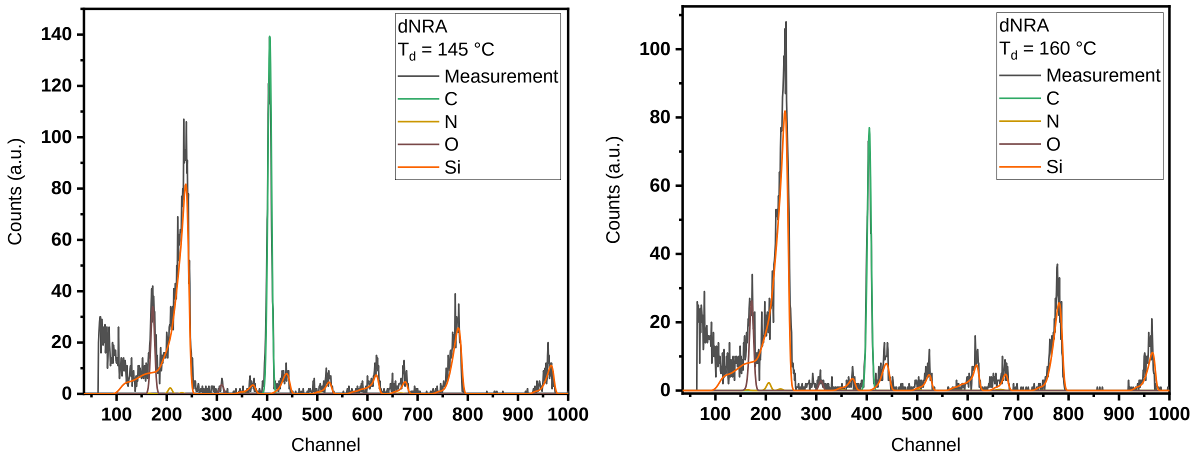

Figure S 41. dNRA spectra of the Cu deposits on Si substrates at a given deposition temperature (T_d). The calibration offset for both measurements is -97.4 keV and energy per channel is 6.85 keV.

S3.References

1 O. V. Dolomanov, L. J. Bourhis, R. J. Gildea, J. A. K. Howard and H. Puschmann, *J. Appl. Crystallogr.*, 2009, **42**, 339–341.

2 G. M. Sheldrick, *Acta Crystallogr. Sect. C Struct. Chem.*, 2015, **71**, 3–8.

3 G. M. Sheldrick, *Acta Crystallogr. Sect. Found. Adv.*, 2015, **71**, 3–8.

4 N. Boysen, B. Misimi, A. Muriqi, J.-L. Wree, T. Hasselmann, D. Rogalla, T. Haeger, D. Theirich, M. Nolan, T. Riedl and A. Devi, *Chem. Commun.*, 2020, **56**, 13752–13755.

5 N. Boysen, T. Hasselmann, S. Karle, D. Rogalla, D. Theirich, M. Winter, T. Riedl and A. Devi, *Angew. Chem. Int. Ed.*, 2018, **57**, 16224–16227.
